# Activation of ventrolateral orbital cortex improves mouse neuropathic pain–induced anxiodepression

**DOI:** 10.1172/jci.insight.133625

**Published:** 2020-10-02

**Authors:** Hai-Yan Sheng, Su-Su Lv, Ya-Qi Cai, Wu Shi, Wei Lin, Ting-Ting Liu, Ning Lv, Hong Cao, Ling Zhang, Yu-Qiu Zhang

**Affiliations:** 1 State Key Laboratory of Medical Neurobiology and MOE Frontiers Center for Brain Science, Department of Translational Neuroscience, Jing’an District Centre Hospital of Shanghai, Institutes of Brain Science, Fudan University, Shanghai, China.; 2 Department of Pathophysiology, School of Basic Medical Sciences, Xinxiang Medical University, Henan, China.; 3 The First Rehabilitation Hospital of Shanghai, Tongji University School of Medicine, Shanghai, China.

**Keywords:** Neuroscience, Depression, Neurological disorders, Pain

## Abstract

Depression and anxiety are frequently observed in patients suffering from neuropathic pain. The underlying mechanisms remained unclear. The ventrolateral orbital cortex (VLO) has attracted considerable interest in its role in antidepressive effect in rodents. In the present study, we further investigated the role of the VLO in the anxiodepressive consequences of neuropathic pain in a chronic constriction injury of infraorbital nerve–induced trigeminal neuralgia (TN) mouse model. Elevated plus maze, open field, forced swimming, tail suspension, and sucrose preference tests were used to evaluate anxiodepressive-like behaviors. The results show that chemogenetic activation of bilateral VLO neurons, especially CaMK2A^+^ pyramidal neurons, blocked the TN-induced anxiodepressive-like behaviors. Chemogenetic and optogenetic activation of VGLUT2^+^ or inhibition of VGAT^+^ VLO neurons was sufficient to produce an antianxiodepressive effect in TN mice. Pharmacological activation of D1-like receptors (D1Rs) but not D2Rs in the VLO significantly alleviated TN-induced depressive-like behaviors. Electrophysiological recordings revealed a decreased excitability of VLO excitatory neurons following neuropathic pain. Furthermore, activation of submedius thalamic nucleus–VLO (Sm-VLO) projection mimicked the antianxiodepressive effect of VLO excitation. Conversely, activation of VLO-periaqueductal gray matter (PAG) projection had no effect on TN-induced anxiodepressive behaviors. This study provides a potentially novel mechanism–based therapeutic strategy for the anxiodepressive consequences of neuropathic pain.

## Abstract 2

Activating excitatory neurons or inhibiting GABAergice neurons of the VLO improved anxiodepressive consequences of neuropathic pain in a trigeminal neuralgia mouse model.

## Graphical abstract

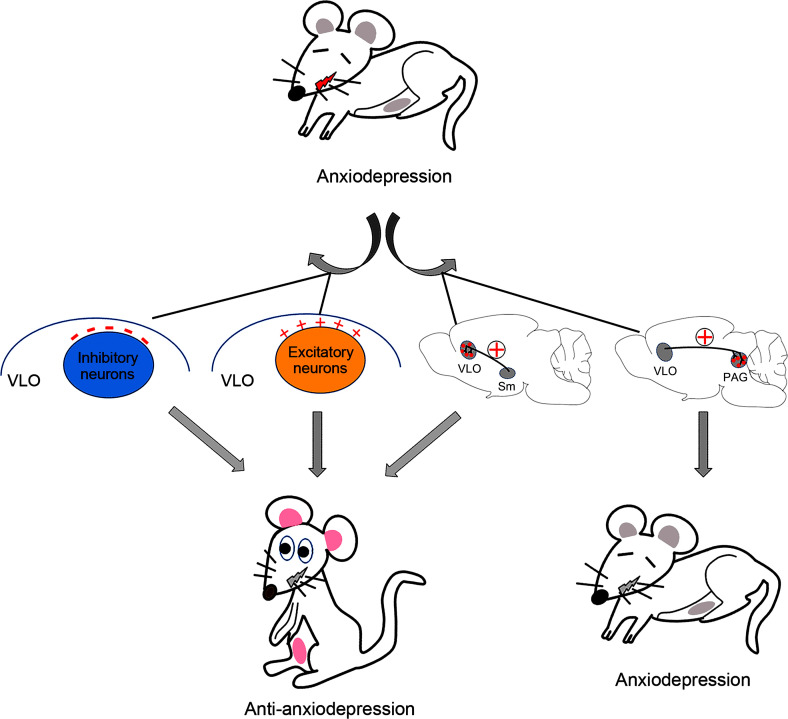

## Introduction

Epidemiology studies indicate that prevalence of chronic pain in the general population is 20%–80%, depending on the differences across the studied populations and the methodology of the studies (1). In patients with chronic pain, depression and anxiety are frequently observed, with prevalence rates ranging from 30% to 50% (2–4). Among people with chronic pain, depression is associated with increased negative thoughts about pain, increased economic burden for patients and their employers, and increased risk of suicide (2, 5). Although the relationship between chronic pain and depression has long been recognized in the clinical setting, so far, relevant brain mechanisms have not been well studied. Animal models provide an important means of understanding the neurobiological basis of depressive consequences of chronic pain. Depressive-like behavior has been observed in several animal models of chronic pain, especially in neuropathic pain models (6, 7).

Advances in biomedical research in recent years indicate that chronic painful disorders share neurobiological aspects, such as neuroplasticity and changes in gene expression with depressive and anxiety disorders (8). A number of studies showed that neuropathic pain–induced mood disorders are sensitive to treatments with anxiolytics and antidepressants (9, 10). Functional imaging studies in humans and animal models of chronic pain reveal structural changes in the corticolimbic brain areas, including anterior cingulate cortex (ACC), medial prefrontal cortex (mPFC), amygdala, hippocampus, and orbitofrontal cortex (OFC), which support emotion, behavior, motivation, and memory functions (11, 12). As a major subdivision of the PFC, the OFC participates directly in negative emotional processing (13–16). Substantial clinical studies found that OFC dysfunction is associated with anxiety (17–19) and depression (18, 20, 21). Treatment with paroxetine, a selective serotonin reuptake inhibitor, in patients with posttraumatic stress disorder can directly improve blood flow to the OFC (22). Moreover, 1 Hz repetitive transcranial magnetic stimulation (rTMS) performed in the right OFC has achieved remission in major depressive disorder patients (23). Thus, it follows that OFC plays an important role in the treatment of anxiety and depression.

Recently, studies have demonstrated that the ventrolateral orbital cortex (VLO), a main subregion of OFC, is highly implicated in the pathogenesis of depression (15, 16, 24, 25) and chronic pain (26). For instance, bilateral intra-VLO injections of 5-aza, a DNA methyltransferase inhibitor, result in a dose-dependent increase in the duration of immobility in the forced swimming test (FST) (27), whereas valproic acid (a mood stabilizer) into the VLO or repeated systemic administration of fluoxetine significantly decreases the immobility time in the FST (28). Our previous study demonstrated that electrolytic or chemical lesions of the bilateral VLO abolished formalin-induced conditioned place aversion, which was considered as a direct reflection of a negative emotion from pain, suggesting that the VLO is involved in pain-related emotion (29). However, the underlying mechanisms are elusive; furthermore, to our knowledge, it has been not reported whether the VLO is implicated in neuropathic pain–induced anxiety and depression.

Here, we provide preclinical evidence that addresses the anxiodepressive consequences of neuropathic pain, using a chronic constriction injury of infraorbital nerve (CION) mouse model that can minimize the effect of limb nerve injury on limb-dependent behaviors to investigate the role of VLO in the regulation of anxiodepressive-like behaviors in trigeminal neuralgia (TN) mice.

## Results

### TN induces anxiodepressive-like behaviors.

**Figure 1:**
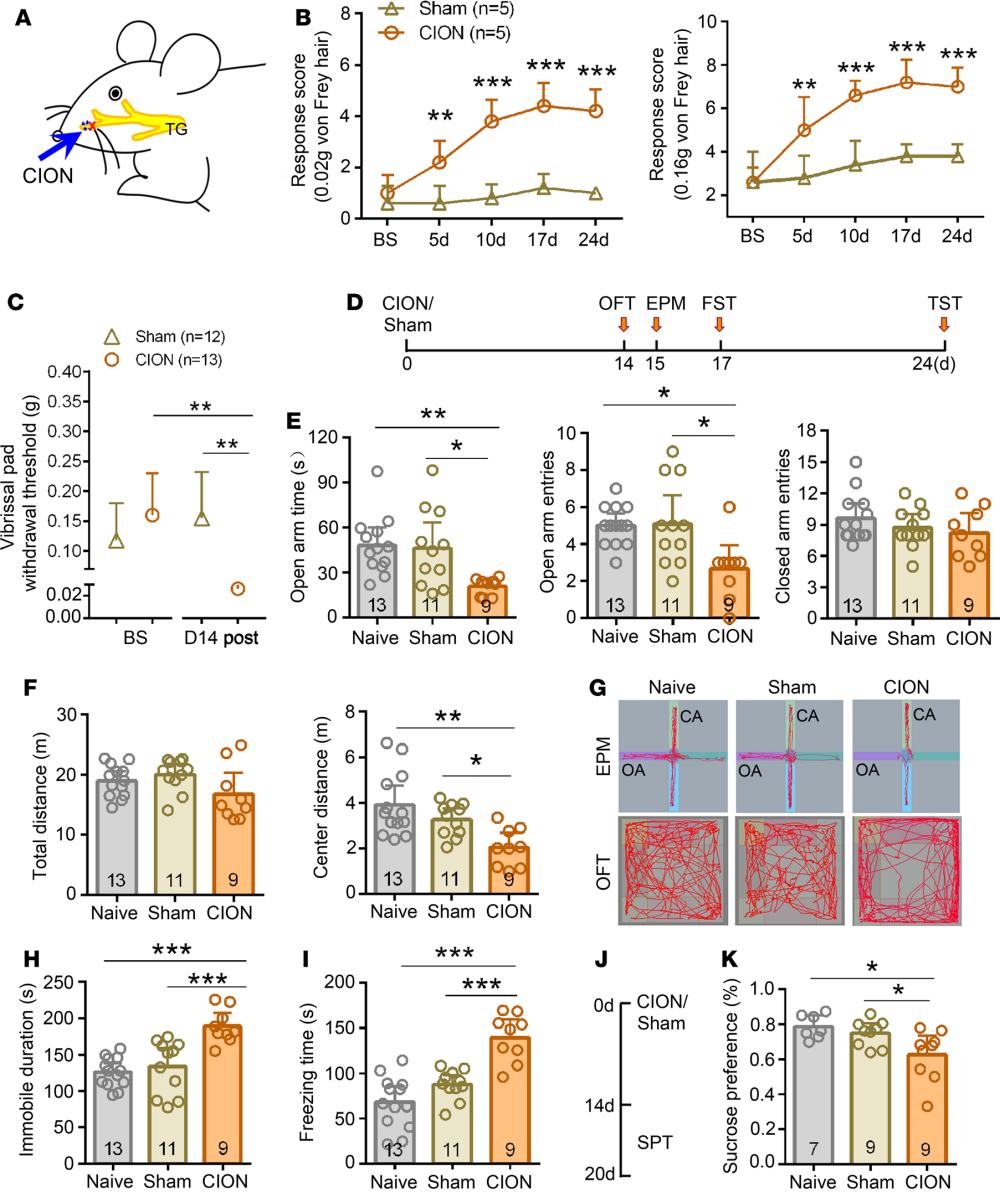
Trigeminal neuralgia induced mechanical allodynia and anxiodepressive-like behaviors in mice. (**A**) Schematic showing chronic constriction injury of infraorbital nerve. CION, arrow points to the ligation site. TG, trigeminal ganglion. (**B**) Following the trigeminal neuralgia (TN) that developed, mechanical stimulation response scores were increased to both 0.02 g (left) and 0.16 g (right) von Frey filaments stimuli on the ipsilateral vibrissa pad. **P < 0.01, ***P < 0.001, 2-way RM ANOVA followed by post hoc Student-Newman-Keuls test; *n* = 5 (both sham and CION). (**C**) Mechanical response threshold of the ipsilateral vibrissa pad decreased markedly on day 14 after CION. **P < 0.01, 1-way ANOVA followed by post hoc Student-Newman-Keuls test; *n* = 12 (sham) and 13 (CION). (**D**) Schematic of the protocol for the experiments in **E**–**I**. (**E**–**G**) The TN mice showed anxiety-like behaviors in elevated plus maze (EPM) test (**E**) and open field test (OFT, **F**). *P < 0.05, **P < 0.01, 1-way ANOVA followed by post hoc Student-Newman-Keuls test; *n* = 13 (naive), 11 (sham), and 9 (CION). (**G**) Example track plots from naive, sham, and CION mice in EPM (above) and OFT (below). (**H** and **I**) The TN mice showed depressive-like behaviors in forced swimming test (FST, **H**) and tail suspension test (TST, **I**). ***P < 0.001, 1-way ANOVA followed by post hoc Student-Newman-Keuls test; *n* = 13 (naive), 11 (sham), and 9 (CION). (**J** and **K**) The TN mice showed depressive-like behaviors in sucrose preference test (SPT). *P < 0.05, 1-way ANOVA followed by post hoc Student-Newman-Keuls test; *n* = 7 (naive), 9 (sham), and 9 (CION).

The TN mouse model was established by chronic CION (Figure 1A) to mimic clinical trigeminal neuropathic pain. After CION, mechanical allodynia developed within 5 days and persisted for at least 24 days in the ipsilateral vibrissa pad. Two-way repeated-measures (RM) ANOVA revealed a significant effect of CION treatment on mechanical stimulation response score (0.02 g, F_1,8_ = 135.40, *P* = 0.0003; 0.16 g, F_1,8_ = 72.0, *P* = 0.001) and interaction between treatment and time (0.02 g, F_4,32_ = 10.21, *P* = 0.0003; 0.16 g, F_4,32_ = 11.88, *P* = 0.0001) compared with the sham group (Figure 1B). A decreased mechanical response threshold in the ipsilateral vibrissa pad was shown in Figure 1C (1-way ANOVA, F_3,23_ = 7.16, *P* < 0.001).

The anxiodepressive-like behavioral tests were conducted from day 14 after CION, when the anxiodepressive-like behaviors occurred steadily according to our previous study (30). As shown in Figure 1, D–G, in the anxiety-related behavioral tests, the TN mice showed less time (1-way ANOVA, F_2,30_ = 5.96, *P* = 0.007) and lower entries (1-way ANOVA, F_2,30_ = 6.24, *P* = 0.005) in the open arm of the elevated plus maze (EPM), and they traveled less distance in the center arena of the open field (OF, 1-way ANOVA, F_2,30_ = 8.07, *P* = 0.002). Since anxiety and depression are often comorbid in clinical patients with chronic pain, we also performed the depression-related behavioral tests, as measured by the FST, tail suspension test (TST), and sucrose preference test (SPT). TN mice exhibited more immobile duration in FST (1-way ANOVA, F_2,30_ = 15.53, *P* < 0.0001, Figure 1H) and freezing time in TST (1-way ANOVA, F_2,30_ = 23.27, *P* < 0.0001) (Figure 1I and Supplemental Video 1; supplemental material available online with this article; https://doi.org/10.1172/jci.insight.133625DS1) relative to the naive or sham group. Another 3 groups of naive, sham, and TN mice were tested in the SPT to examine anhedonia, and decreased sucrose preference was observed in TN mice (Figure 1, J and K; 1-way ANOVA, F_2,22_ = 5.50, *P* = 0.001). These results suggest that CION-induced TN mice displayed anxiodepressive-like behaviors. There was no difference between the naive group and the sham group in these behavioral tests.

### Activation of VLO neurons produces an antianxiodepressive effect in TN mice.

**Figure 2:**
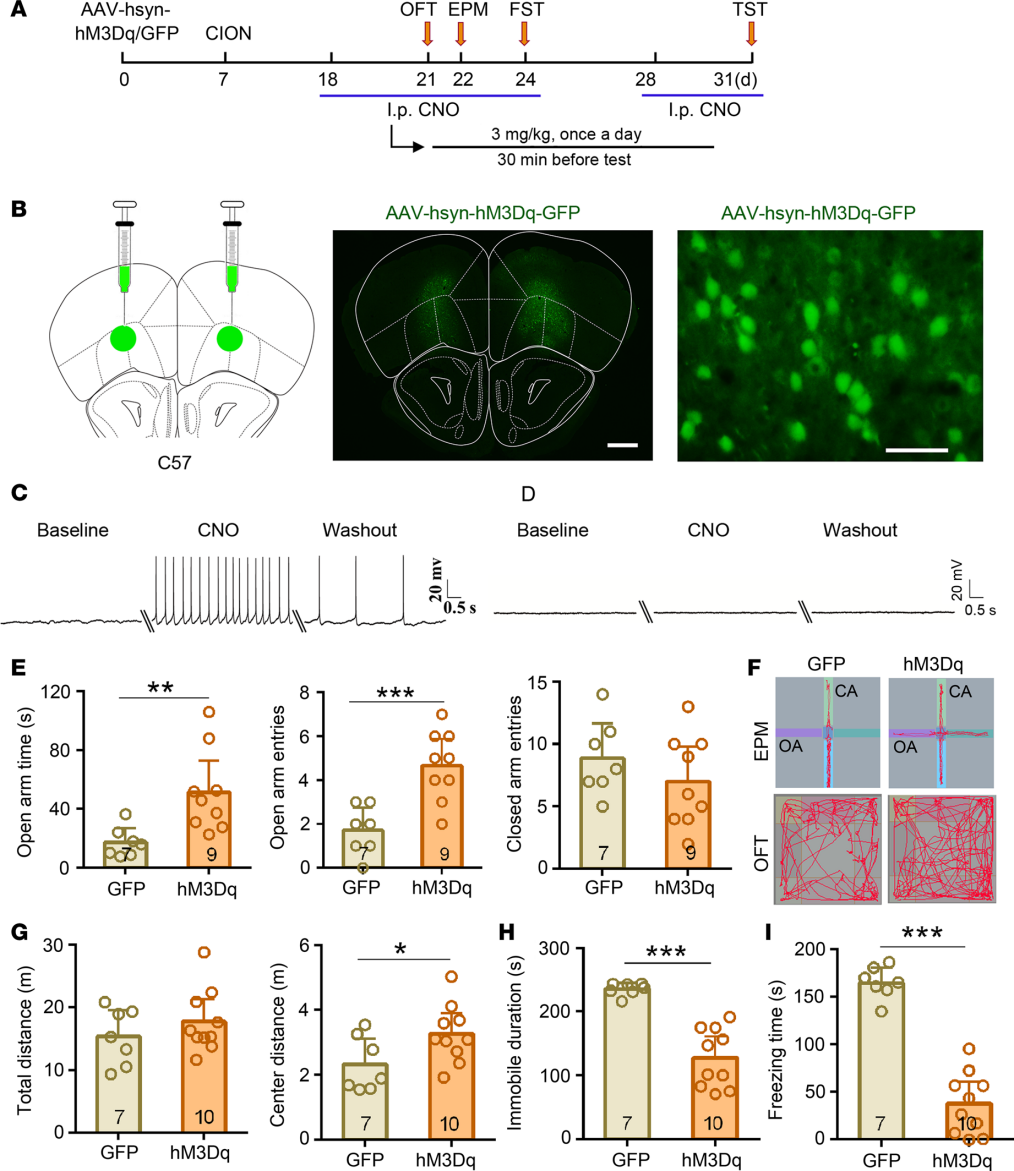
Activation of VLO neurons induced an antianxiodepressive effect in TN mice. (**A**) Schematic of the protocol for the experiments in **E**–**I**. (**B**) Schematic and photomicrograph of coronal section showing AAV-hsyn-hM3Dq-GFP injection into the bilateral VLO. Scale bar: 500 μm for low magnification, 50 μm for high magnification. (**C** and **D**) Examples showing that the effects of bath CNO (500 nM) on GFP^+^ neurons firing in VLO slices from mice injected with AAV-hM3Dq-GFP (**C**) and AAV-GFP (**D**). (**E**–**I**) Activation of bilateral VLO neurons by chemogenetic manipulation produced an antianxiodepressive effect in EPM and OFT (**E**–**G**), FST (**H**), and TST (**I**). *P < 0.05, **P < 0.01, ***P < 0.001, 2-sided Student’s *t* test; *n* = 7 (GFP) and 9–10 (hM3Dq).

Our previous study indicated that the VLO was involved in pain-related negative emotion (29). To explore the role of the VLO in neuropathic pain–induced anxiodepressive-like behaviors, we activated VLO neurons by chemogenetic manipulation. The adeno-associated virus–encoding (AAV-encoding) engineered Gq-coupled hM3D receptor (AAV-hsyn-hM3Dq-GFP) or AAV-hsyn-GFP (control) was bilaterally injected into the VLO, and clozapine-N-oxide (CNO, 3 mg/kg) was i.p. administrated to achieve specific activation of VLO neurons expressing hM3D receptor (Figure 2, A and B). The efficacy of hM3Dq-mediated excitation was confirmed by whole-cell patch clamp recordings in VLO slices. Action potential (AP) firing was induced by CNO (500 nM) in VLO neurons expressing hM3Dq (Figure 2C), but not GFP (Control, Figure 2D). The behavioral test showed that, following the CNO, TN mice exhibited typical antianxiety-like behaviors, marked by more open arm time and open arm entries in the EPM test, as well as more center distance in the OF test (OFT) (Figure 2, E–G). There was no difference between 2 groups for closed arm entries in the EPM test (Figure 2E) and total travel distance in the OFT (Figure 2G), indicating that the phenotypic differences were not due to hyperactivity. Consistently, antidepressive-like behaviors were induced by activating VLO neurons in TN mice. Both the immobile duration in FST and freezing time in TST were lower in the hM3D group than in the GFP group (Figure 2, H and I). We further confirmed the antianxiodepressive effect of the VLO activation by pharmacological intervention. Microinjection of DL-Homocysteic acid (HCA, 30 nmol per side), a glutamate replacement, into the bilateral VLO led to a significant antianxiodepressive effect in TN mice compared with the normal saline (NS) controls (Supplemental Figure 1, A–G).

We also examined the effect of VLO activation on anxiety and depression levels in normal mice. No statistical difference was detected among the 3 groups: naive, hM3D plus CNO, and hM3D plus NS groups (Supplemental Figure 2, A–E).

### Inhibition of VLO neurons has no effect on TN-induced anxiodepressive-like behaviors.

**Figure 3:**
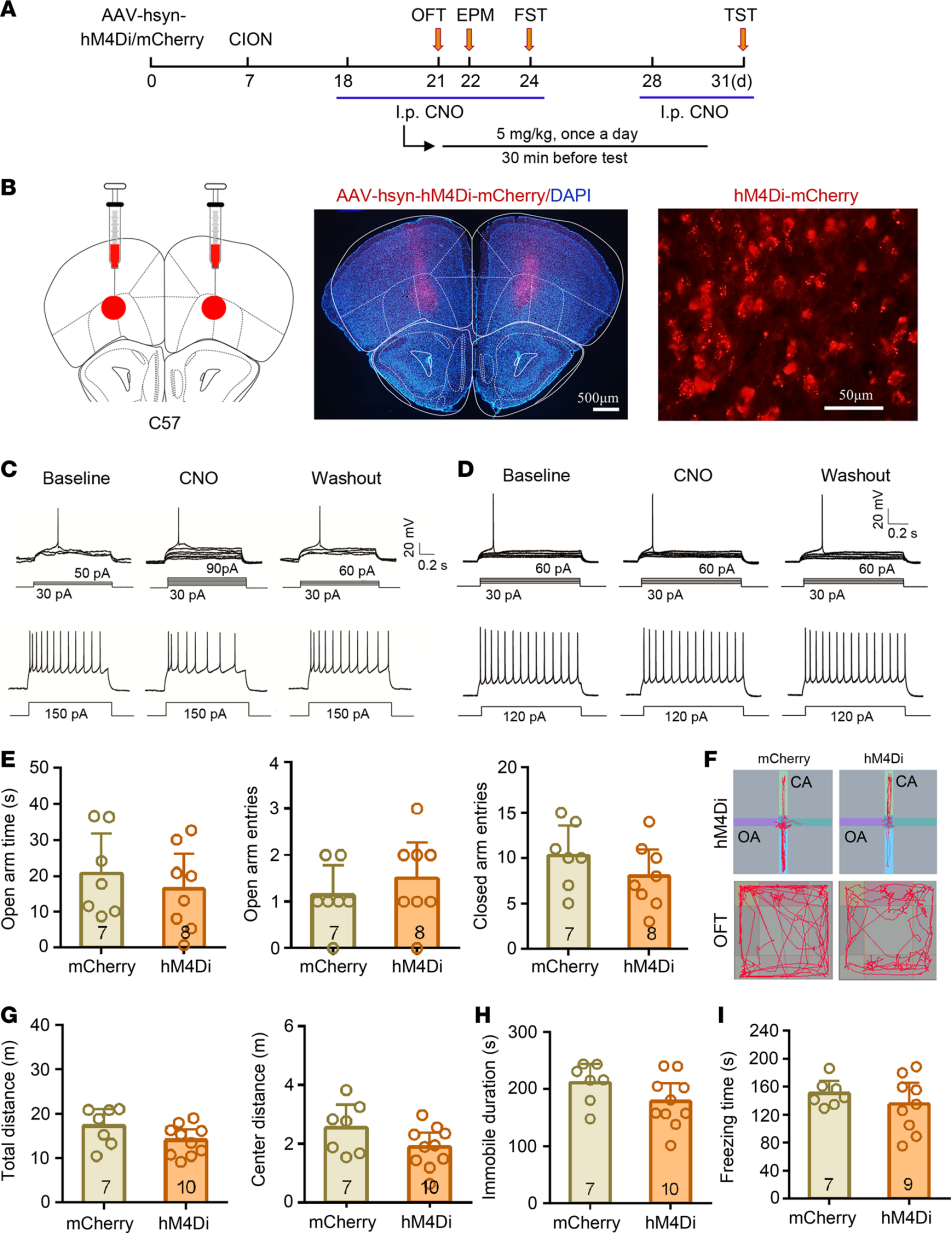
Inhibition of VLO neurons did not affect the anxiodepressive-like behaviors in TN mice. (**A**) Schematic of the protocol for the experiments in **E**–**I**. (**B**) Schematic and photomicrograph of coronal section showing AAV-hsyn-hM4Di-mCherry injection into the bilateral VLO. Scale bar: 500 μm for low magnification, 50 μm for high magnification. (**C** and **D**) Examples showing that bath CNO (500 nM) increased the injected currents threshold to evoke action potentials (APs) and decreased action potential firing frequencies in AAV-hM4Di-mCherry injection mice (**C**), but not in AAV-mCherry injection ones (**D**). (**E**–**I**) Inhibition of bilateral VLO neurons failed to change the anxiodepressive-like behaviors of TN mice in EPM and OFT (**E**–**G**), FST (**H**), and TST (**I**). Two-sided Student’s *t* test; *n* = 7 (mCherry) and 8–10 (hM4Di).

We next inhibited VLO neurons chemogenetically with bilateral injection of AAV-encoding engineered Gi-coupled hM4D receptor (AAV-hsyn-hM4Di-mCherry) (Figure 3, A and B). Whole-cell patch clamp recordings in VLO slices showed that CNO incubation increased the injected currents threshold to evoke APs and decreased AP firing frequencies in hM4Di-expressing VLO neurons (Figure 3C), but not in mCherry-expressing ones (Figure 3D), confirming the hM4Di-mediated inhibitory effect on VLO neurons. As shown in Figure 3, E–I, TN-induced anxiodepressive-like behaviors were not changed by VLO inhibition. No significant difference was seen in all the anxiodepressive-like behavioral tests between the hM4Di treatment and mCherry control mice. We also examined the effect of VLO inhibition in normal mice; neither AAV-hsyn-hM4Di nor AAV-CaMK2A-hM4Di in bilateral VLO neurons altered the anxiodepressive level in normal mice (Supplemental Figure 3 and 4).

### TN decreases the excitability of VLO excitatory neurons.

**Figure 4:**
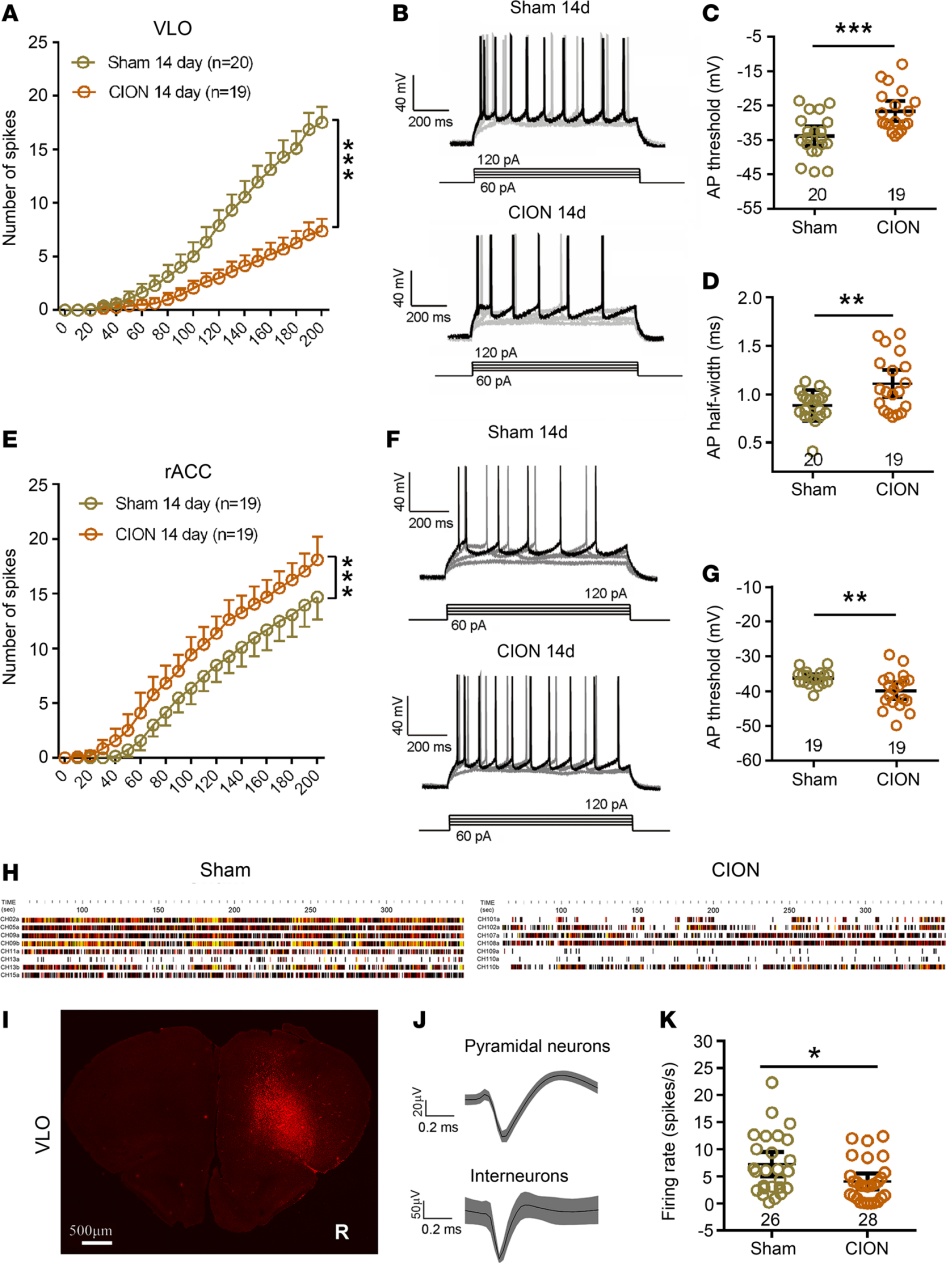
The excitability of VLO excitatory pyramidal neurons decreased in TN mice. (**A**) Number of spikes induced by injected currents in VLO CaMK2A^+^ pyramidal neurons from sham and CION mice. ***P < 0.001, 2-way ANOVA followed by post hoc Student-Newman-Keuls test; *n* = 20 (sham) and 19 (CION; cells). (**B**) Examples of AP responses to positive current steps recording from CaMK2A^+^ pyramidal neurons in the VLO from sham and CION mice. (**C** and **D**) Quantification of AP thresholds (**C**) and half-width (**D**) in VLO CaMK2A^+^ pyramidal neurons from sham and CION mice. **P < 0.01, ***P < 0.001, 2-sided Student’s *t* test; *n* = 20 (sham) and 19 (CION; cells). (**E**) Number of spikes induced by injected currents in rACC CaMK2A^+^ pyramidal neurons from sham and CION mice. ***P < 0.001, 2-way ANOVA followed by post hoc Student-Newman-Keuls test; *n* = 19 (both sham and CION; cells). (**F**) Examples of AP responses to positive current steps recording from CaMK2A^+^ pyramidal neurons in the rACC from sham and CION mice. (**G**) Quantification of AP thresholds in rACC CaMK2A^+^ pyramidal neurons from sham and CION mice. **P < 0.01, 2-sided Student’s *t* test; *n* = 19 (both sham and CION; cells). (**H**) Examples of multiple channel recordings in vivo during TST in 1 sham and 1 CION mice. (**I**) Photomicrograph of coronal section showing the site of multiple-channel electrode implantation in unilateral VLO (contralateral to the CION). Scale bar: 500 μm. (**J**) Example showing that VLO pyramidal neurons has a long duration compared with interneurons in multiple channel electrophysiological recordings in vivo. (**K**) The spontaneous firing rate of VLO pyramidal neurons in TN mice was lower during the TST. *P < 0.05, 2-sided Student’s *t* test; *n* = 26 (sham) and 28 (CION; cells).

To address whether TN altered the excitability of VLO excitatory neurons, the biophysical characteristics of APs were examined in contralateral VLO CaMK2A^+^ pyramidal neurons using whole-cell patch clamp recordings. We applied 10 pA step depolarizing current pulses to evoke APs. The input-output curves appeared right-shifted at day 14 after CION, indicating that fewer APs were elicited by depolarizing current steps as compared with sham mice (Figure 4, A and B; 2-way ANOVA, F_1,37_ = 171.5, *P* < 0.0001). In CION mice, the VLO CaMK2A^+^ pyramidal neurons had a more depolarized firing threshold and a greater half-width of APs (Figure 4, C and D). No significant differences in resting membrane potential, input resistance, membrane capacitance, and time constant (Tau) were identified between sham and CION groups (Supplemental Figure 5, A–D). These data suggest that the intrinsic excitability of VLO excitatory neurons was decreased at the single-neuron level after TN. On the contrary, in CaMK2A^+^ pyramidal neurons of the rostral ACC (rACC) adjacent to the VLO, the input-output curves appeared left-shifted with a lower depolarized firing threshold at day 14 after CION (2-way ANOVA, F_1,36_ = 127.8, *P* < 0.0001), suggesting that the excitability of rACC excitatory neurons was increased (Figure 4, E–G). No significant differences in resting membrane potential, input resistance, membrane capacitance, and Tau were identified between sham and CION groups (Supplemental Figure 5, E–H).

To further address whether the spontaneous activity of VLO pyramidal neurons was altered in TN-induced depressive-like behavior, we performed in vivo multichannel electrophysiological recordings at day 15 after CION. We identified pyramidal neurons and interneurons based on spike duration and firing rate (31). Compared with interneurons, pyramidal neurons have a longer duration and lower firing rate. A total of 80 VLO neurons was recorded in 4 sham and 3 CION mice. Most of the neurons (54 of 80) are pyramidal neurons: 26 from sham and 28 from CION mice. During the TST, VLO pyramidal neurons from CION mice had a significantly lower in vivo spontaneous firing rate than those from sham mice (Figure 4, H–K, and Supplemental Figure 5I).

### Activation of VLO glutamatergic neurons contributes to the antianxiodepressive effect in TN mice.

**Figure 5:**
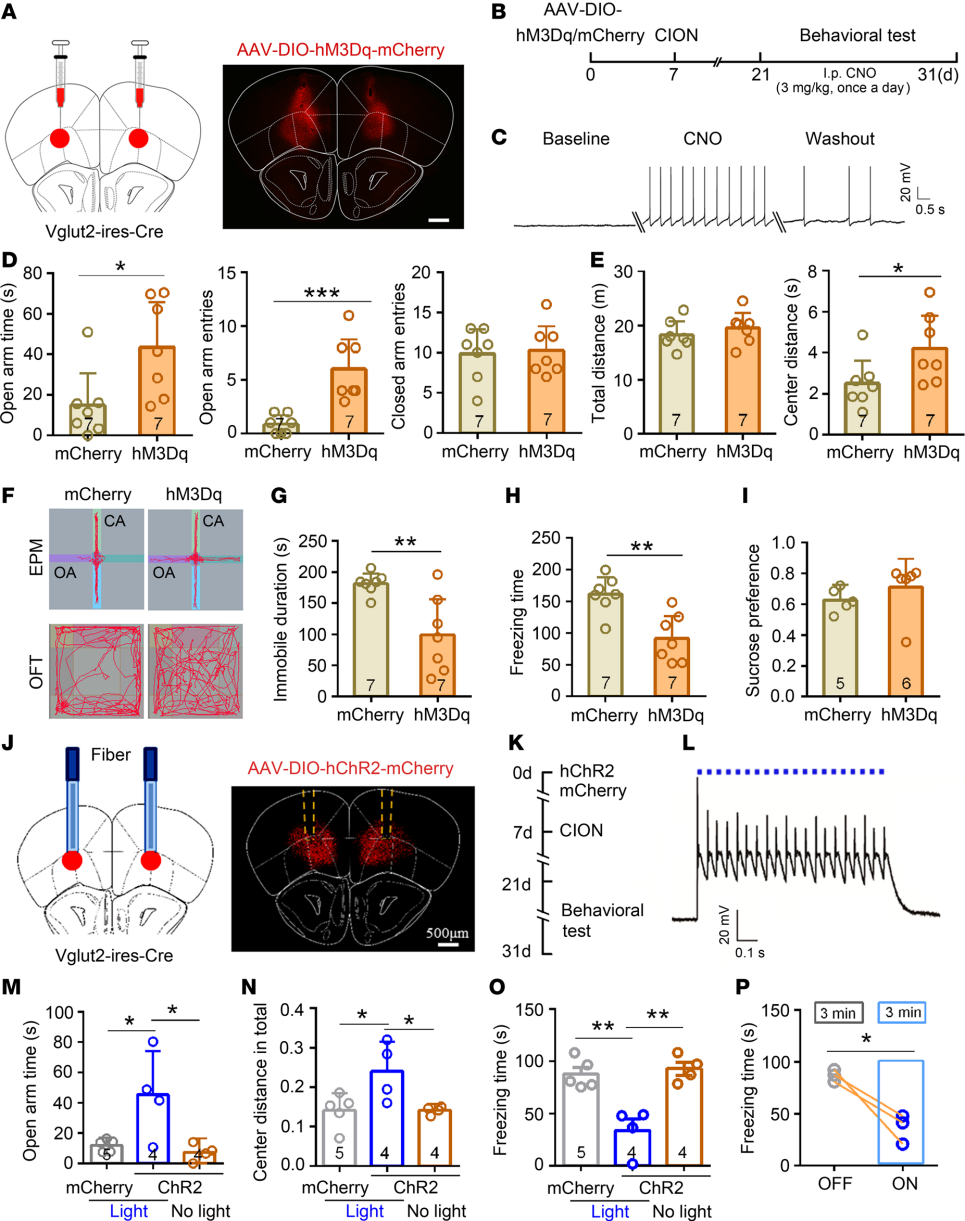
Selective activation of VLO glutamatergic neurons produced an antianxiodepressive effect in TN mice. (**A**) Schematic and photomicrograph of coronal section showing AAV-DIO-hM3Dq-mCherry injection into the bilateral VLO of Vglut2-IRES-Cre mice. Scale bar: 500 μm. (**B**) Schematic of the protocol for experiments **D**–**I**. (**C**) An example showing that bath CNO (500 nM) evoked action potentials (APs) in VGLUT2^+^ neurons expressing hM3Dq-mCherry. (**D**–**H**) Activation of bilateral VLO VGLUT2^+^ neurons by chemogenetic manipulation produced an antianxiodepressive effect in EPM and OFT (**D**–**F**), FST (**G**), and TST (**H**). *P < 0.05, **P < 0.01, ***P < 0.001, 2-sided Student’s *t* test; *n* = 7 (both mCherry and hM3Dq). (**I**) Activation of bilateral VLO VGLUT2^+^ neurons by chemogenetic manipulation did not affect sucrose presence in SPT (2-sided Student’s *t* test; *n* = 5 mCherry and 6 hM3Dq). (**J**) Schematic and photomicrograph of coronal section showing the site of optical fiber implantation and AAV-DIO-hChR2-mCherry injection into the bilateral VLO of Vglut2-IRES-Cre mice. Scale bar: 500 μm. (**K**) Schematic of the protocol for experiments in **M**–**P**. (**L**) Patch clamp recording in VLO slice showing that action potentials induced through blue light stimulation (473 nm, 5 mW, 20 Hz) on VLO VGLUT2^+^ neurons expressing ChR2-mCherry. (**M**–**O**) Optogenetic activation of bilateral VLO VGLUT2^+^ neurons produced an antianxiodepressive effect in EPM (**M**), OFT (**N**), and TST (**O**). *P < 0.05, **P < 0.01, 1-way ANOVA followed by post hoc Student-Newman-Keuls test; *n* = 5 (mCherry-light), 4 (ChR2-light), and 4 (ChR2-no light). (**P**) Blue light stimulation of VLO VGLUT2^+^ neurons expressing ChR2-mCherry reduced the freezing time in TST with lights on for 3 minutes. *P < 0.05, 2-sided paired *t* test; *n* = 3 (ChR2-light off and light on).

We next sought which cell population was implicated in the antianxiodepressive effect in TN mice. The functional role of VGLUT2^+^ neurons in the VLO was tested with the aid of a Cre-recombinase–enabled chemogenetic activating system. The Cre-recombinase–dependent AAV (AAV-DIO-hM3Dq) was bilaterally injected into the VLO in Vglut2-IRES-Cre mice (Figure 5, A and B). I.p. injection of CNO was then used to specifically activate VGLUT2^+^ neurons within the VLO. The efficacy of hM3Dq-mediated excitation was confirmed with VLO slice recordings (Figure 5C). A typical antianxiodepressive effect was obtained in the DIO-hM3Dq group relative to DIO-mCherry controls following i.p. injection of CNO in TN mice. The antianxiety effect was marked by significant increase in open arm time and open arm entries in the EPM test, as well as center distance in the OFT (Figure 5, D–F). The antidepressive effect was marked by a remarkable decrease in both immobile duration in the FST and freezing time in the TST (Figure 5, G and H). However, activation of VLO glutamatergic neurons failed to improve anhedonia of TN mice in the SPT (Figure 5I), consistent with the previous study from Ortega et al. (32). This suggests that an increase in excitatory activity of VLO neurons may be mainly antagonistic to the despairing phenotype rather than the anhedonic phenotype of depressive-like behaviors.

This antianxiodepressive effect of activating VLO glutamatergic neurons was further confirmed by optogenetic manipulation through intra-VLO injection of Cre-dependent ChR2-mCherry (AAV-DIO-ChR2) in Vglut2-IRES-Cre mice (Figure 5, J and K). Activation of glutamatergic neurons by optogenetic stimulation was verified in the VLO slice recordings (Figure 5L). Optogenetic activation (473 nm, 20 Hz, 25 ms, stimulation for 3 minutes) of VLO glutamatergic neurons led to a significant antianxiodepressive effect in the OFT, EPM test, and TST (Figure 5, M–P and Supplemental Video 2).

### Activation of CaMK2A^+^ pyramidal neurons in the VLO is implicated in the antianxiodepressive effect in TN mice.

**Figure 6:**
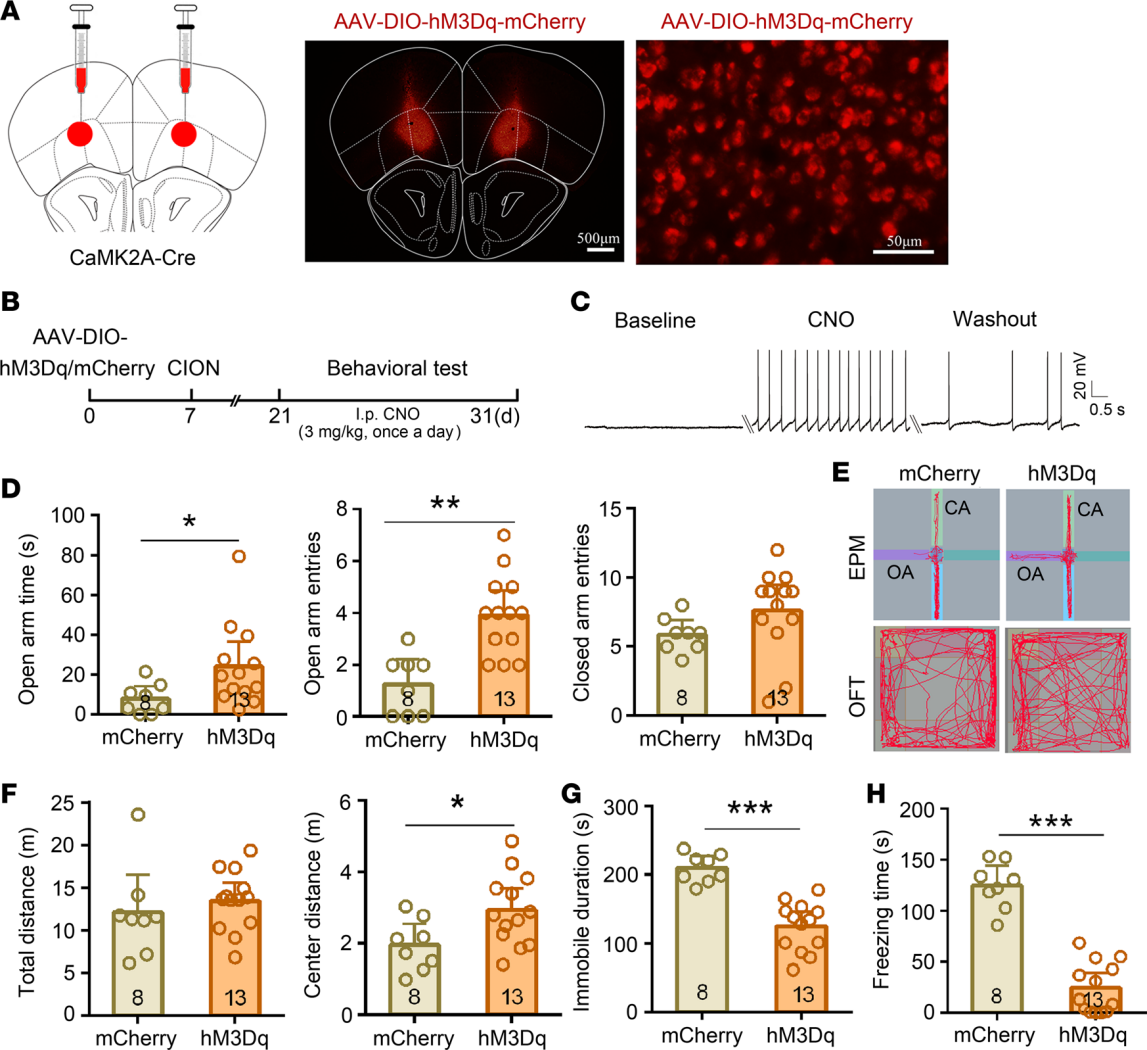
Selective activation of VLO CaMK2A^+^ neurons induced an antianxiodepressive effect in TN mice. (**A**) Schematic and photomicrograph of coronal section showing AAV-DIO-hM3Dq-mCherry injection into the bilateral VLO of CaMK2A-Cre mice. Scale bar: 500 μm for low magnification, 50 μm for high magnification. (**B**) Schematic of the protocol for the experiments in **D**–**H**. (**C**) An example showing that bath CNO (500 nM) evoked action potentials (APs) in hM3Dq-mCherry injection mice. (**D**–**H**) Activation of bilateral VLO CaMK2A^+^ neurons by chemogenetic manipulation produced an antianxiodepressive effect in EPM and OFT (**D**–**F**), FST (**G**), and TST (**H**). *P < 0.05, **P < 0.01, ***P < 0.001, 2-sided Student’s *t* test; *n* = 8 (mCherry) and 13 (hM3Dq).

To further verify the role of VLO excitatory neurons in the antianxiodepressive effect, we achieved selective activation of VLO CaMK2A^+^ excitatory neurons by bilateral intra-VLO injection of AAV-DIO-hM3Dq in CaMK2A-Cre mice (Figure 6, A and B). Whole-cell patch clamp recordings in VLO slices showed that AP firing was induced by CNO in CaMK2A^+^ neurons expressing hM3Dq (Figure 6C). The behavioral tests showed that the hM3Dq group were significantly increased in open arm time (2-sided Student’s *t* test, t_[19]_ = 2.11, *P* < 0.05) and open arm entries (2-sided Student’s *t* test, t_[19]_ = 4.18, *P* = 0.0005) in the EPM test, but it did not affect the closed arm entries (Figure 6, D and E). Meanwhile, the center distance of the mice in the OFT was also increased (2-sided Student’s *t* test, t_[19]_ = 2.435, *P* = 0.025; Figure 6F). In the FST and TST, activating CaMK2A^+^ VLO neurons produced a robust antidepressive effect (2-sided Student’s *t* test; FST, t_[19]_ = 6.13, *P* < 0.0001; TST t_[19]_ = 9.24, *P* < 0.0001; Figure 6, G and H). This result further indicates that VLO excitatory neurons mediated the antianxiodepressive effect in TN mice.

### Inhibition of GABAergic neurons in the VLO produces the antianxiodepressive effect in TN mice.

**Figure 7:**
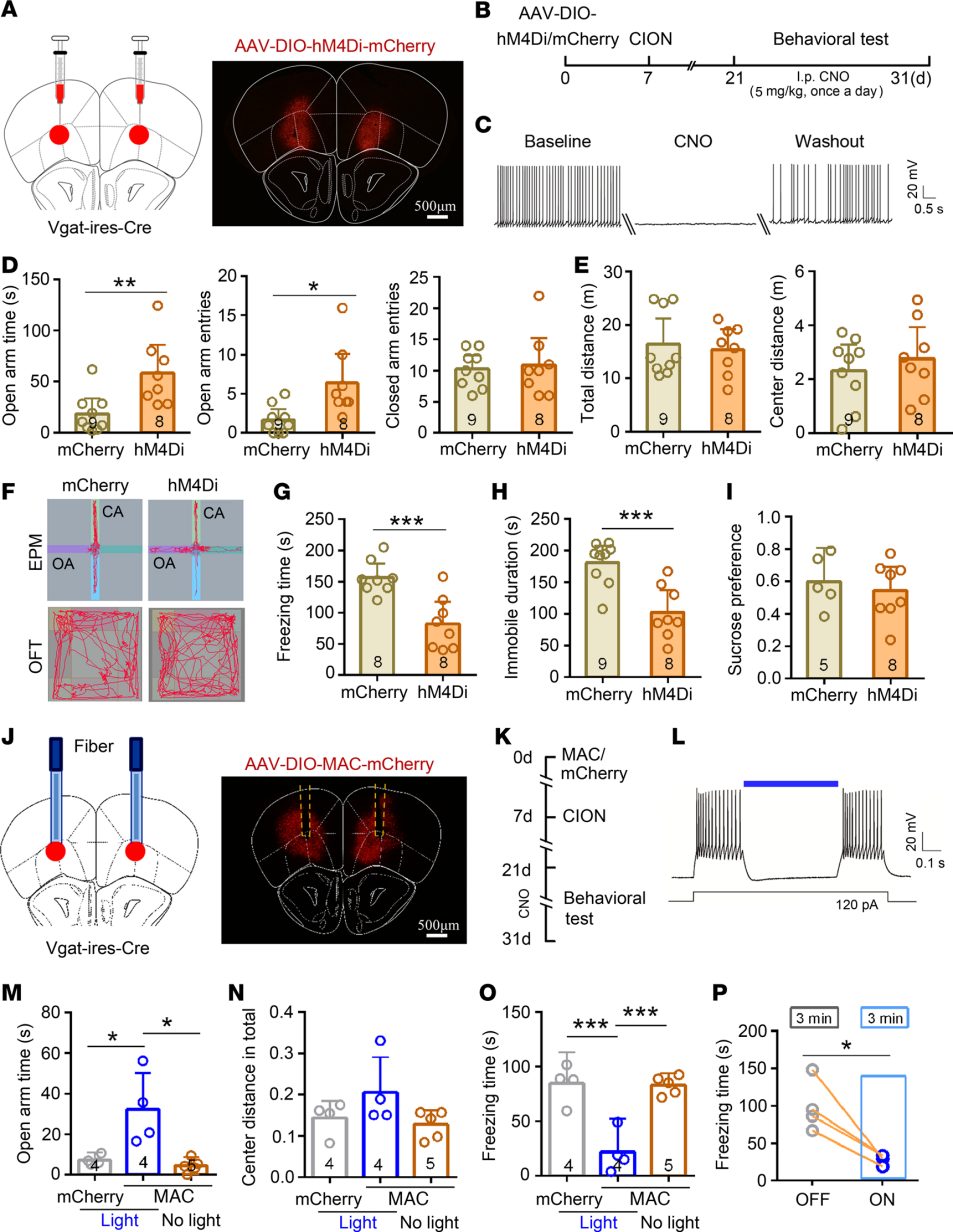
Selective inhibition of VLO GABAergic neurons produced the an antianxiodepressive effect in TN mice. (**A**) Schematic and photomicrograph of coronal section showing AAV-DIO-hM4Di-mCherry injection into the bilateral VLO of Vgat-IRES-Cre mice. Scale bar: 500 μm. (**B**) Schematic of the protocol for experiments in **D**–**I**. (**C**) An example showing that bath CNO (500 nM) suppressed action potentials (APs) firing in VGAT^+^ neurons expressing hM4Di-mCherry. (**D**–**H**) Inhibition of bilateral VLO VGAT^+^ neurons by chemogenetic manipulation produced an antianxiodepressive effect in EPM (**D** and **F**), FST (**G**), and TST (**H**), but not in OFT (**E**). *P < 0.05, **P < 0.01, ***P < 0.001, 2-sided Student’s *t* test; *n* = 8–9 (mCherry) and 8 (hM4Di). (**I**) Inhibition of bilateral VLO VGAT^+^ neurons by chemogenetic manipulation did not affect sucrose preference in SPT (2-sided Student’s *t* test; *n* = 5 mCherry and 8 hM4Di). (**J**) Schematic and photomicrograph of coronal section showing the site of optical fiber implantation and AAV-DIO-MAC-mCherry injection into the bilateral VLO of Vgat-IRES-Cre mice. Scale bar: 500 μm. (**K**) Schematic of the protocol for experiments in **M**–**P**. (**L**) Patch clamp recording in VLO slice showing that APs were suppressed through blue light stimulation (473 nm, 5 mW, continuous) on VLO VGAT^+^ neurons expressing MAC-mCherry. (**M**–**O**) Optogenetic inhibition of bilateral VLO VGAT^+^ neurons induced an antianxiodepressive effect in EPM (**M** and **N**) and TST (**O**). *P < 0.05, ***P < 0.01, 1-way ANOVA followed by post hoc Student-Newman-Keuls test; *n* = 4 (mCherry-light), 4, (MAC-light) and 5 (MAC-no light). (**P**) Light stimulation of VLO VGAT^+^ neurons expressing MAC-mCherry reduced the freezing time in TST with lights on for 3 minutes. *P < 0.05, 2-sided paired *t* test; *n* = 4.

We also selectively silenced VLO GABAergic neurons by bilateral intra-VLO infusion of Cre-recombinase–dependent AAV (AAV-DIO-hM4Di) in Vgat-IRES-Cre mice (Figure 7, A and B). The efficacy of hM4Di-mediated inhibition was confirmed with VLO slice recordings (Figure 7C). Similar results to activating VLO glutamatergic neurons were obtained in the EPM test, FST, TST, and SPT. Inhibition of VLO GABAergic neurons produced a significantly antianxiodepressive effect (Figure 7, D–I). Optogenetic inhibition of VLO GABAergic neurons was also performed by intra-VLO injection of Cre-dependent MAC-mCherry (AAV-DIO-MAC) in Vgat-IRES-Cre mice (Figure 7, J and K). The blocking effect on VLO GABAergic neurons by optogenetic stimulation was examined in the VLO slice recordings (Figure 7L). Selective inhibition of VLO GABAergic neurons through continuous light (473 nm, stimulation for 3 minutes) further confirmed the results obtained in chemogenetic manipulation (Figure 7, M–P, and Supplemental Video 3).

### Activation of D1-like but not D2-like dopamine receptors in the VLO produces the antidepressive effect in TN mice.

**Figure 8:**
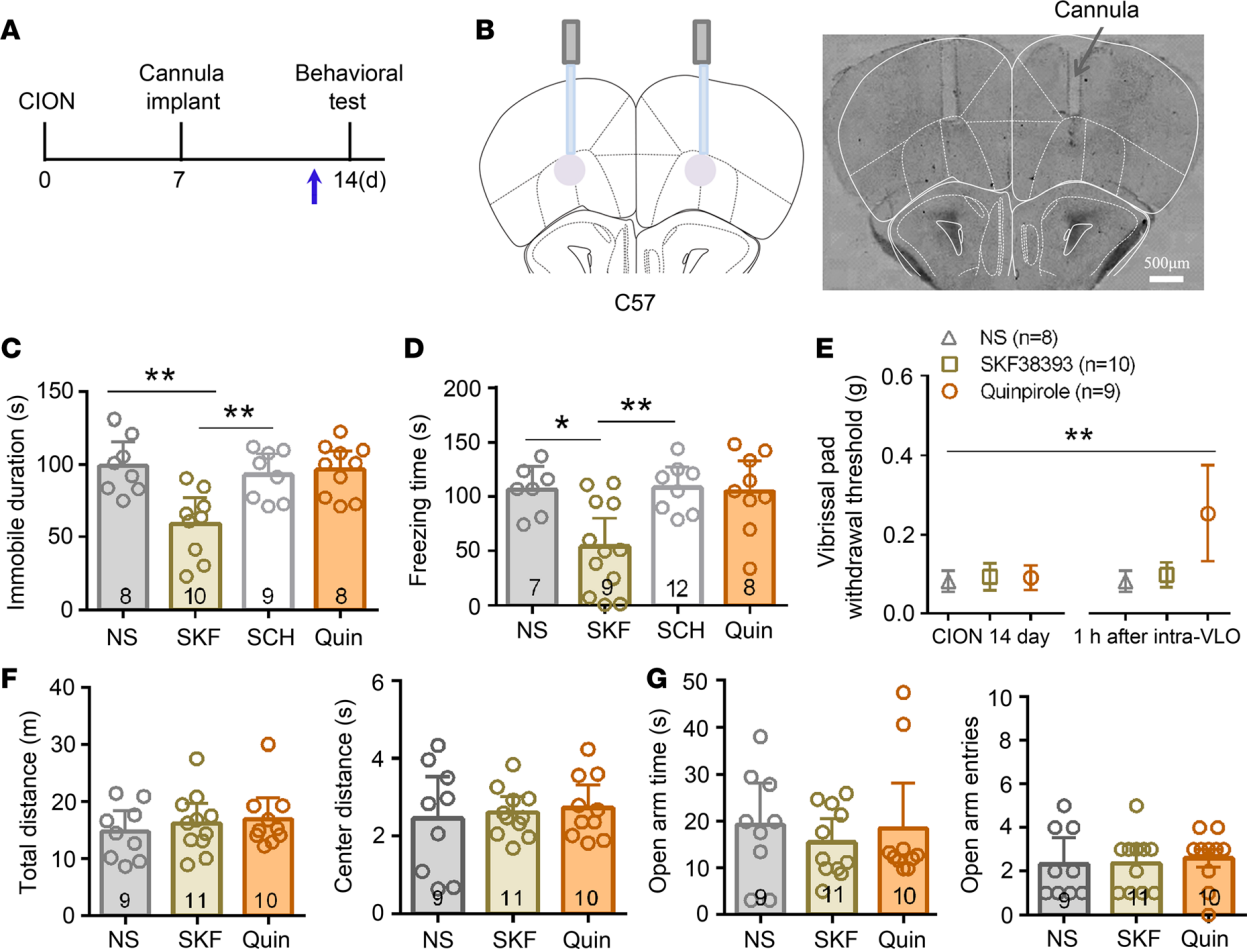
Effects of D1Rs and D2R agonists into the VLO on TN-induced anxiodepressive-like behaviors and mechanical allodynia. (**A**) Schematic of the protocol for experiments in **C**–**G**. (**B**) Schematic and photomicrograph of coronal section showing cannula placement in the bilateral VLO in mice. Scale bar: 500 μm. (**C** and **D**) Microinjection of D1R agonist SKF38393 (3 μg, per side) but not D2R agonist quinpirole (1 μg, per side) into the bilateral VLO led to a significant antidepressive effect in FST (**C**) and TST (**D**), which can be blocked by D1Rs antagonist SCH23390 (3 μg, per side) in TN mice. *P < 0.05, **P < 0.01, 1-way ANOVA followed by post hoc Student-Newman-Keuls test; *n* = 8 (NS), 10 (SKF38393), 9 (SCH23390), and 8 (quinpirole; FST, **C**); *n* = 7 (NS), 9 (SKF38393), 12 (SCH23390), and 8 (quinpirole; TST, **D**). (**E**) Microinjection of D2R agonist quinpirole but not D1R agonist SKF38393 attenuated TN-induced mechanical allodyina. **P < 0.01, 1-way ANOVA followed by post hoc Student-Newman-Keuls test; *n* = 8 (NS), 10 (SKF38393), and 9 (quinpirole). (**F** and **G**) Neither D1R agonist SKF38393 nor D2R agonist quinpirole into the bilateral VLO influenced TN-induced anxiety–like behaviors in OFT (**F**) and EPM (**G**). One-way ANOVA; *n* = 9 (NS), 11 (SKF38393), and 10 (quinpirole).

The dopaminergic system is a candidate neurotransmitter system thought to be involved in depression (33). Both excitatory pyramidal neurons and GABAergic inhibitory interneurons in the frontal cortex including VLO express D1-like receptors (D1Rs) and D2Rs (34–36). Thus, we further tested the effects of pharmacologically activating D1Rs or D2Rs on TN-induced anxiodepression. Microinjection of D1R agonist SKF38393 (3 μg, per side) into the bilateral VLO led to a significant antidepressive effect in FS and TS tests, which could be blocked by D1Rs antagonist SCH23390 (3g, per side) in TN mice (1-way ANOVA; FST, F_3,31_ = 8.14, *P* = 0.0004; TST, F_3,32_ = 6.57, *P* = 0.001). Conversely, D2R agonist quinpirole (1 μg, per side) into the bilateral VLO had no effect on TN-induced depressive-like behaviors (Figure 8, A–D). By contrast, TN-induced mechanical allodynia was attenuated by D2R agonist quinpirole but not D1R agonist SKF38393 (Figure 8E; 1-way ANOVA, F_5,48_ = 7.4, *P* < 0.0001), consistent with previous studies that D2R agonists but not D1R agonists in the VLO produced an analgesic effect in normal rats and spared sciatic nerve injury animals (37, 38). There was no difference among the groups in OF (Figure 8F) and EPM tests (Figure 8G), indicating that activation of D1Rs and D2Rs did not affect spontaneous locomotor activity and TN-induced anxiety–like behaviors.

### Activation of Sm-VLO projection pathway contributes to the antianxiodepressive effect in TN mice.

**Figure 9:**
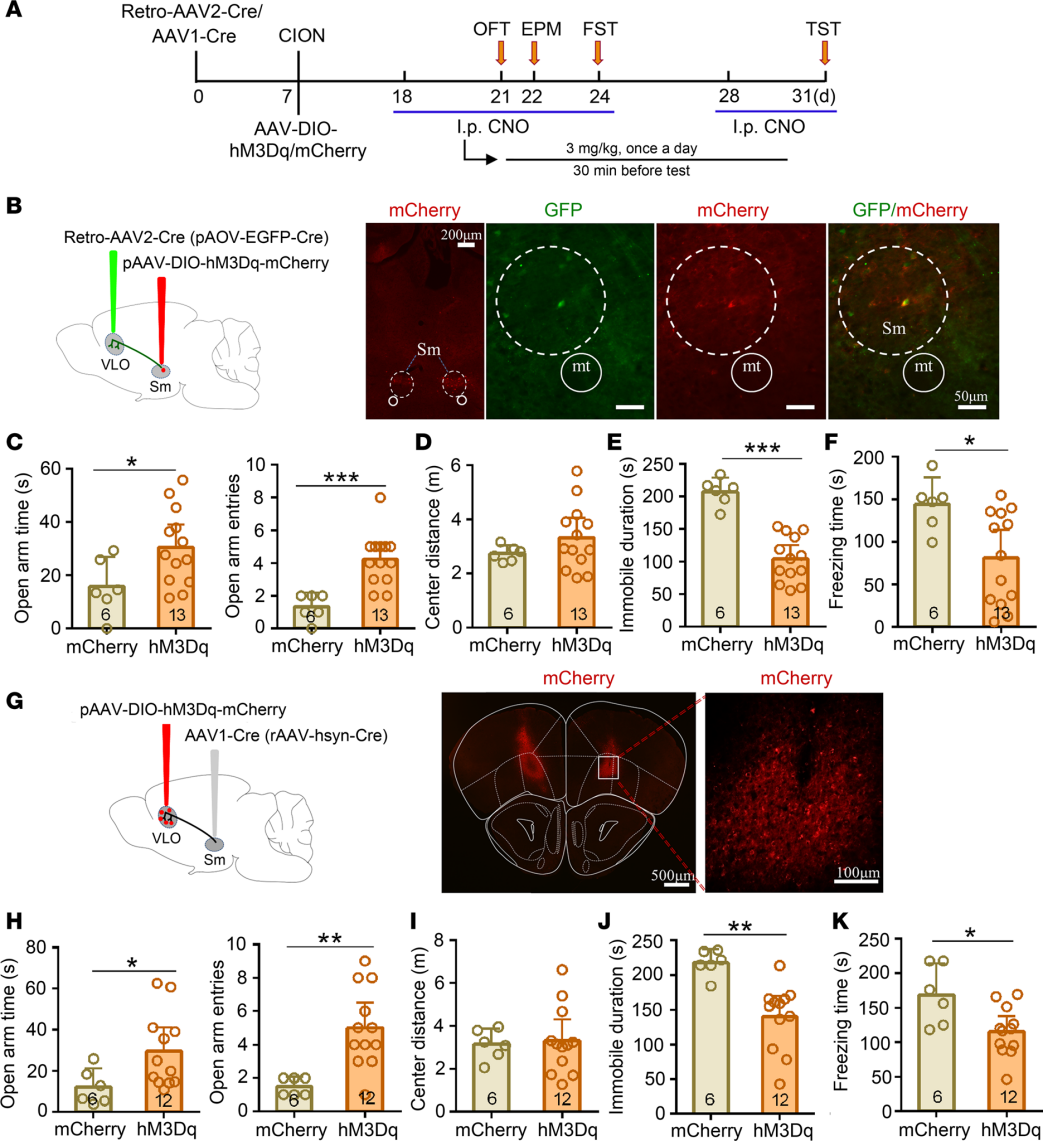
Chemogenetic activation of Sm-VLO projection pathway resulted in the antianxiodepressive effect in TN mice. (**A**) Schematic of the protocol for experiments in **B**–**K**. (**B**) Sagittal schematic diagrams showing retro-AAV2-CRE-GFP injection into the bilateral VLO and AAV-DIO-hM3Dq-mCherry (or AAV-DIO-mCherry) injection into the bilateral Sm in mice. Photomicrograph of coronal section showing Cre-dependent mCherry^+^ signals in the bilateral Sm (low magnification) and both retrograde labeled and Cre-dependent mCherry double-labeled neurons in the Sm (higher magnification). Scale bar: 200 μm for low magnification, 50 μm for high-magnification. (**C**–**F**) Activation of the Sm-VLO projection pathway by chemogenetic manipulation produced an antianxiodepressive effect in EPM (**C**), FST (**E**), and TST (**F**), but not in OFT (**D**). *P < 0.05, ***P < 0.001, 2-sided Student’s *t* test; *n* = 6 (mCherry) and 13 (hM3Dq). (**G**) Sagittal schematic diagrams and photomicrograph of coronal section showing AAV1-Cre injection into the bilateral Sm and AAV-DIO-hM3Dq-mCherry (or AAV-DIO-mCherry) injection of the bilateral VLO in mice. Scale bar: 500 μm for low magnification, 100 μm for high magnification. (**H**–**K**) Chemogenetic activation of the VLO neurons receiving projection from Sm produced an antianxiodepressive effect in EPM (**H**), FST (**J**), and TST (**K**), but not in OFT (**I**). *P < 0.05, **P < 0.01, 2-sided Student’s *t* test; *n* = 6 (mCherry) and 12 (hM3Dq).

The VLO mainly received the projections from the ipsilateral submedius thalamic nucleus (Sm) (39, 40). We verified these results by retrograde tracing with fluorescent RetroBeads IX to label the mouse Sm-VLO projection pathway (Supplemental Figure 6A). To address whether Sm-VLO projection was involved in VLO activation–mediated antianxiodepressive effect, we used a Cre-recombinase–enabled chemogenetic activation system, via injection of the retrograde virus–expressed CRE recombinase (pAOV-GFP-CRE) into the bilateral VLO and a Cre-recombinase–dependent AAV (pAAV-DIO-hM3Dq-mCherry) into the bilateral Sm (Figure 9A). The retrograde labeled soma and Cre-dependent mCherry neurons in the Sm are visible (Figure 9B). The Sm-VLO projecting neurons were specifically activated via i.p. CNO; the mice exhibited significant antianxiety effects in the EPM test and antidepressive effects in the FST and TST relative to the mCherry control mice (Figure 9, C–F). It has been reported that the Sm-VLO pathway is involved in nociceptive modulation (35). We further observed that activation of Sm-VLO projecting neurons attenuated TN-induced mechanical allodyina (Supplemental Figure 6B).

To further verify the antianxiodepressive effect of Sm-VLO projection, we next injected recombinant AAV1-Cre (rAAV-hSyn-Cre) into the bilateral Sm and the AAV-DIO-hM3Dq into the bilateral VLO for specific infection of VLO postsynaptic neurons with hM3Dq or mCherry (Figure 9, G and H). AAV1-Cre from transduced presynaptic neurons can effectively and specifically drive Cre-dependent transgene expression in selected postsynaptic neuronal targets (41, 42). Thus, the VLO neurons receiving Sm neuron axonal projections were specifically activated via the chemogenetic activating system; the mice exhibited a similar antianxiodepressive effect in the EPM test, FST, and TST (Figure 9, I–K). These data indicate that the Sm-VLO projection pathway mediated the antianxiodepressive effect in TN mice.

### Activation of VLO-vlPAG projection pathway failed to improve the anxiodepressive effect in TN mice.

**Figure 10:**
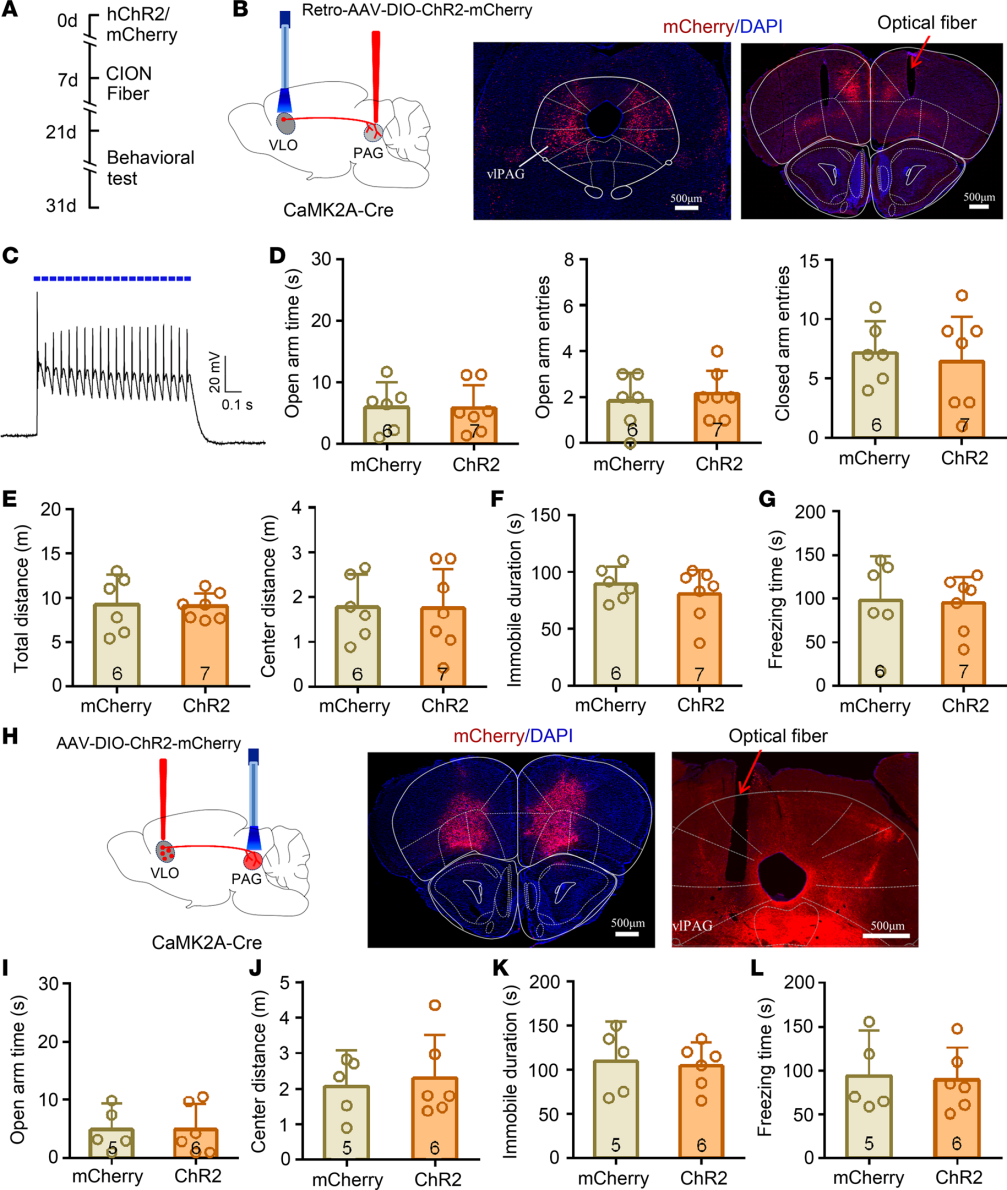
Optogenetic activation of VLO-vlPAG projection pathway had no effect on TN-induced anxiodepressive-like behaviors. (**A**) Schematic of the protocol for experiments in **C**–**M**. (**B**) Sagittal schematic diagrams and photomicrograph of coronal section showing retro-AAV-DIO-ChR2-mCherry injection into the bilateral vlPAG of CaMK2A-Cre mouse (left) and CaMK2A^+^ neurons expressing ChR2-mCherry in the VLO (right). Scale bar: 500 μm. (**C**) An example showing that action potentials induced through blue light stimulation (473 nm, 5 mW, 20 Hz) on VLO CaMK2A^+^ neurons expressing ChR2-mCherry. (**D**–**G**) Optogenetic activation of VLO CaMK2A^+^ neurons projecting to vlPAG did not affect TN-induced anxiodepressive effect in EPM (**D**), OFT (**E**), FST (**F**), and TST (**G**). Two-sided Student’s *t* test; *n* = 6 (mCherry) and 7 (ChR2). (**H**) Sagittal schematic diagrams and photomicrograph of coronal section showing injection of AAV-DIO-ChR2-mCherry into the bilateral VLO of CaMK2A-Cre mouse (left) and CaMK2A^+^ terminal expressing ChR2-mCherry in the vlPAG (right). Scale bar: 500 μm. (**I**–**L**) Optogenetic activation of the VLO-vlPAG excitatory projection had no effect on TN-induced anxiodepressive effect in EPM (**I**), OFT (**J**), FST (**K**), and TST (**L**). Two-sided Student’s *t* test; *n* = 5 (mCherry) and 6 (ChR2).

Anatomical studies have established that, in rats and cats, the VLO projects bilaterally to the lateral or ventrolateral parts of the periaqueductal gray matter (vlPAG) (35), a region that has been implicated intensively in descending modulation of nociception (43). In the present study, we further confirmed the VLO-containing CaMK2A^+^ pyramidal neurons that project to the PAG via injection of the Cre-recombinase–dependent adeno-associated retrograde virus (pAAV-EF1a-DIO-hChR2[H134]-mCherry) into the bilateral vlPAG of CaMK2A-Cre mice (Figure 10, A and B). The VLO-vlPAG projecting neurons were specifically activated via optogenetic stimulation (473 nm, 20 Hz, 25 ms) for 3 minutes. As shown in Figure 10, C–G, TN-induced anxiodepressive-like behaviors were not changed by blue light stimulation. No significant difference was seen in all the anxiodepressive-like behavioral tests between the ChR2-treated and mCherry control mice.

To further verify the effect of VLO-vlPAG excitatory projection in TN-induced anxiodepressive-like behaviors, Cre-dependent ChR2-mCherry (AAV-DIO-ChR2) were injected into the bilateral VLO of CaMK2A-Cre mice (Figure 10H). Optogenetic activation of CaMK2A^+^ terminal–expressing ChR2 in the vlPAG had no effect on TN-induced anxiodepressive-like behaviors (Figure 10, I–L, and Supplemental Figure 6, C and D). Given that VLO-vlPAG projection has been demonstrated to mediate descending inhibition of pain (35, 44), we also examined the effect of activating VLO-vlPAG excitatory projection on TN-induced mechanical allodynia. As expected, optogenetic activation of the VLO-vlPAG pathway significantly reduced mechanical allodyina (Supplemental Figure 6E).

## Discussion

In the present study, we provide insight into the role of regulating VLO neuronal activity in the anxiodepressive consequences of neuropathic pain. Our results showed that either activation of excitatory neurons or inactivation of inhibitory neurons in the VLO improved neuropathic pain–induced anxiodepression. Activation of the Sm-VLO projection but not VLO-vlPAG projection contributed to the antianxiodepressive actions of VLO neuronal activation. Additionally, we observed that D1R agonist but not D2R agonist in the VLO was sufficient to produce a rapid antidepressive effect. This is a potentially unique study investigating the functional significance of VLO neuronal activity in neuropathic pain–induced anxiodepression. We propose an antidepressive strategy for anxiodepressive consequences of neuropathic pain by manipulating VLO neuronal activity.

### Involvement of VLO neuronal activity in neuropathic pain–induced anxiodepression.

As mentioned above, one of the important findings in the current study is that increase in excitatory activity of VLO neurons produced a robust antianxiodepressive effect in neuropathic pain mice, when selectively activating glutamatergic neurons or inhibiting GABAergic neurons. However, activation of VLO neurons does not change the anxiodepressive level in normal mice, indicating that manipulating VLO neuronal excitatory activity is sufficient to ameliorate anxiodepressive-like behaviors in neuropathic pain conditions, leaving the emotional level of normal mice stable. Furthermore, increasing OFC function or directly stimulating this brain area has also been reported to improve stress-induced anxiety/depression disorder. For example, when paroxetine, was used to treat posttraumatic stress disorder, local cerebral blood flow and brain function in the OFC were increased (22). Increasing the OFC gray matter volume protected against symptoms of anxiety through increased optimism (45). TMS of the right OFC effectively remitted the symptoms of patients with major depression (23).

Chronic pain is usually accompanied by aberrant neuronal activity, such as hyperactivity, in multiple cortical and subcortical regions, including the ACC and amygdala (46). However, the present results showed that the intrinsic excitability of VLO pyramidal neurons is decreased on day 14 after CION, when the anxiodepressive-like behaviors occurred steadily. Multichannel electrophysiological recordings in vivo showed that VLO pyramidal neurons from CION mice had a significantly lower spontaneous firing rate during the TST, suggesting a decreased excitatory activity of VLO neurons in neuropathic pain accompanied by anxiodepressive consequence. This decreased excitability of VLO excitatory neurons in mice with neuropathic pain may partly explain that emotional disorders such as anxiety and depression are associated with decreased metabolic activity (17) and reduced OFC volume in clinical patients (20, 47–49) and experimental primates (50). Consistently, Wang et al. demonstrated that the intrinsic excitability of excitatory pyramidal neurons in layers 2 and 3 of prelimbic cortex (PL), a subregion of mPFC, is decreased in adult (6–8 weeks) Sprague-Dawley rats with CFA inflammatory pain. Activation of PL excitatory pyramidal neurons resulted in an obviously anxiolytic effect in inflammatory pain animals (51). Conversely, Matos et al. observed an increased neuronal excitability of layers 2 and 3 pyramidal neurons in the mPFC and ACC of young (3 weeks) Long-Evans rats with spared nerve injury (52). This contradiction may be related to the differences in strains and ages of experimental animals, chronic pain models, and subregions of mPFC.

Despite the decreased neuronal activity and cortical volume (or cortical thickness) of the VLO in chronic pain accompanied by emotional disorder, inhibition of VLO neurons did not affect TN-induced anxiodepressive level. In particular, inhibiting VLO neurons and even selectively inhibiting VLO excitatory neurons failed to induce anxiodepressive-like behaviors in naive mice. We therefore speculate that the decreased excitability and spontaneous firing rate of VLO neurons may not contribute to the initiation of anxiodepressive-like behaviors; it is more likely to be a consequence of neuropathic pain and anxiodepression.

Consistent with previous reports that the excitability of ACC pyramidal neurons is enhanced by injury of sciatic nerve or its branches (7, 52, 53), as a positive control, we observed the enhanced intrinsic excitability of ACC pyramidal neurons in a trigeminal nerve injury model. Given that inhibition of the ACC hyperactivity was sufficient to alleviate the aversive and anxiodepressive-like consequences of neuropathic pain, the pain-related aversion and anxiodepression may be underpinned by ACC hyperactivity (7). Thus, the ACC hyperactivity and VLO hypoactivity may play different roles in modulation of neuropathic pain–induced anxiodepressive-like consequences.

### D1R agonist but not D2R agonist ameliorates TN-induced anxiodepression.

There is evidence that the dopaminergic system plays an important role in anxiodepressive and nociceptive modulation in various brain areas (35, 36, 54). D1Rs and D2Rs were highly expressed in the frontal cortex, including VLO excitatory pyramidal neurons and GABAergic inhibitory interneurons (34–36, 55). Therefore, we examined the effects of activating D1Rs and D2Rs in the VLO on TN-induced anxiodepression. The results showed that D1R agonist SKF38393 into the VLO led to a marked antidepressive effect but did not attenuate allodynia in TN mice. In contrast, D2R agonist quinpirole did not affect TN-induced anxiodepression but produced an analgesic effect, mimicking the analgesic effect of D2R agonist into the VLO in rat acute pain (37) and neuropathic pain (38). The absence of an antidepressive effect of D2R activation in the VLO is consistent with clinical research from Larisch et al. that the closest correlation between alleviation from major depression and D2R changes was found in the striatum and cingulate gyrus, but not in superior frontal gyrus and OFC (56). A recent study from Duman’s laboratory showed that activation of *Drd1-* but not *Drd2-*expressing pyramidal neurons in the mPFC of *Drd1-* and *Drd2*-Cre recombinase mice produced rapid and long-lasting antidepressive effects (36), suggesting an important role of frontal cortex *Drd1–*expressing excitatory neurons in antidepression. It is known that D1R links to Gs/Gq proteins that stimulate AC-cAMP-PKA/PLC-PKC pathways to facilitate excitatory synaptic transmission (57). Stimulation of D1Rs but not D2Rs enhanced NMDA receptor–mediated EPSCs (58, 59) and excitability of PFC pyramidal neurons (60). Thus, it is reasonable to presume that the antidepressive effect of D1R agonist may be achieved by increasing the activity of VLO excitatory pyramidal neurons via D1Rs. Further study is needed to determine the source of VLO dopaminergic projections, the types of neurons expressing D1Rs and D2Rs, and the role of these neurons in the regulation of anxiodepressive disorder.

### Activation of Sm-VLO but not VLO-vlPAG projection produces an antianxiodepressive effect in TN mice.

From the peripheral receptor to the cerebral cortex, multidimensional sensory, motivational, and affective aspects of pain have focused on 2 parallel systems: lateral pain pathway, being thought to transmit information of noxious stimulus characters, and medial pain pathway, being proposed to process information relating to pain-related negative emotion (61). Sm in the ventromedial thalamus is an important thalamic relay nucleus of medial pain pathway. Previous tracing studies have established that, in rats and cats, the VLO receives major projections from the thalamic Sm (39) and projects to the PAG (62), a region that has been implicated intensively in descending modulation of nociception (48). A series of studies from Tang’s laboratory have demonstrated that the Sm, VLO, and PAG constitute a pain modulatory pathway, activation of which facilitates the descending inhibitory system and suppresses nociceptive inputs in the spinal cord and trigeminal nucleus (35). A recent study demonstrated that dorsal Sm VGLUT2^+^ neurons formed a single axon arbor exclusively within the VLO (40). The emotional and aversive aspects of the VLO function require the integration of information from Sm (63). It is reasonable to presume that the direct thalamic-cortical projection from the Sm to VLO is involved in anxiodepressive or antianxiodepressive effect. Indeed, by combining retrograde and anterograde virus expressed with chemogenetic manipulation, we demonstrated that activation of the Sm-VLO pathway produced an obviously antianxiodepressive effect in TN mice.

The PAG is thought to be a fundamental region that was involved in defensive and antinociceptive behaviors. The vlPAG, a subdivision of the PAG, has been shown to participate in not only analgesia and defensive behavior in response to threat, but also stress-induced anxiodepressive-like behaviors (64, 65). The frontal cortex, including the VLO, has been shown to preferentially target ventral PAG, particularly the vlPAG (35, 66), which is further confirmed by the retrograde and anterograde virus tracking in the present study. Intriguingly, optogenetic activation of the VLO-vlPAG excitatory pathway failed to alleviate anxiodepressive-like behaviors in TN mice, despite its analgesic effect. This result seems to contradict the role of PAG in anxiodepression. A reasonable explanation is that vlPAG integrates several inputs from various emotion-related brain regions and sends out projections to a large variety of regions, such as central nucleus of the amygdala (CeA) and lateral habenula (66), which are highly involved in anxiodepressive mechanisms (67, 68).

In addition to the vlPAG, VLO also projects to the mPFC (16, 69, 70) and amygdala (69–71), and it connects to the lateral habenula via the striatum and pallidum; in turn, the habenula projects to the raphe nuclei and ventral tegmental area to produce an antianxiodepressive effect (16). Considering the important role of the mPFC, amygdala, and lateral habenula in pain-related anxiodepression and the connections between the VLO with them, it is likely that the neural network of these structures is implicated in VLO-mediated antianxiodepressive effect in TN mice.

In conclusion, the present study reveals an important role of the VLO in antagonizing anxiodepressive consequences of chronic pain. The increase in excitatory neuronal activity within the VLO and activation of the Sm-VLO projection pathway contribute to this process. This study provides a therapeutic strategy for the anxiodepressive consequences of neuropathic pain.

## Methods

### Animals

C57BL/6J WT, CAMK2A-Cre (The Jackson Laboratory, 005359), Vglut2-IRES-Cre (The Jackson Laboratory, 016963), and Vgat-IRES-Cre (The Jackson Laboratory, 016962) and non–Cre-expressing littermate mice (6–12 weeks, 20–25g) were used in this study (male C57BL/6J, both male and female transgenic strains). All mice were bred in a temperature- and humidity-controlled room on a 12-hour light/dark cycle with access to food and water ad libitum. The body weight and sexes of transgenic animals were assigned to different treatment groups randomly. All of the behavioral testing and electrophysiological recording experiments described herein were performed by experimenters who were blind to the treatments.

### TN surgery

The TN model was produced by chronic constriction injury of the unilateral infraorbital nerve via an intraoral approach, as described previously (30). Mice were anesthetized with sodium pentobarbital (50 mg/kg, i.p.); then, the skin of the left cheek was disinfected with iodine volt. The head was fixed, and the mouth was kept open during the operation. A 0.5-cm surgical incision was made at 0.1 cm proximal to the first molar along the left gingivobuccal margin. The left infraorbital nerve was slightly ligated with 4-0 chromic gut ligatures to produce the CION model. Sham-operated mice received only nerve exposure but not ligation. All surgical procedures were performed aseptically.

### AAV and stereotaxic injection

Mice were anesthetized with sodium pentobarbital (50 mg/kg, i.p.) and then placed in a stereotaxic apparatus. The skull plane was adjusted to make sure the bregma and lambda were at a horizontal level. The virus was injected through a glass microelectrode attached to a microinjection pump (Hamilton, Nanoliter 2010 injector, World Precision Instruments Inc.) at a guaranteed rate 30 nL/min. The microelectrode injection needle was not withdrawn until 10 minutes after the end of infusion, allowing the virus to diffuse sufficiently.

AAV vector expressing the hM3Dq or hM4Di receptor (AAV-hsyn-hM3Dq-GFP, 1.59 × 10^13^ vg/mL; AAV-hsyn-hM4Di-mCherry, 2.66 × 10^13^ vg/mL, 220 nL per side, OBiO Technology Co., Ltd.) was microinjected bilaterally into the VLO (anterioposterior [AP], +2.5 mm; mediolateral [ML], ±1.0 mm; dorsoventral [DV], –1.8 mm — according to the mouse atlas of Paxinos and Watson) of C57BL/6J mice.

To selectively express the hM3Dq receptor in excitatory neurons (CaMK2A^+^), glutamatergic (VGLUT2^+^) neurons, or hM4Di receptor in GABAergic (VGAT^+^) neurons of the VLO, the Cre-dependent hM3Dq-AAV (AAV-DIO-hM3Dq-mCherry, 9.00 × 10^13^ vg/mL, OBiO Technology Co., Ltd.) or Cre-dependent hM4Di-AAV (AAV-DIO-hM4Di-mCherry, 1.45 × 10^13^ vg/mL, OBiO Technology Co., Ltd.) was administrated to the bilateral VLO of CaMK2A-Cre, Vglut2-IRES-Cre, and Vgat-IRES-Cre mice, respectively. The DIO-mCherry-AAV was microinjected as a control virus in each of these groups. The DIO-hM3Dq or DIO-hM4Di was injected into the VLO of non–Cre-expressing littermate mice as vector injection controls. All injection volumes were 220 nL per side.

To activate the Sm-VLO projection pathway, a retrograde AAV (AAV-hSyn-GFP-2A-Cre, 5.96 × 10^13^ vg/mL, 300 nL per side, OBiO Technology Co., Ltd. Shanghai) was preinjected into the bilateral VLO. One week later, the Cre-dependent hM3Dq-AAV (150 nL per side) was microinjected into the bilateral Sm (AP, –1.33 mm; ML, ±0.38 mm; DV, –4.5 mm). Also, AAV1-Cre (rAAV-hSyn-Cre-WPRE-pA, 1.07 × 10^13^ vg/mL, 150 nL per side, BrainVTA) was injected into the bilateral Sm before the VLO microinjection of AAV-DIO-hM3Dq-mCherry; the injection of the 2 virus were separated by 1 week (41, 42).

In order to achieve the optogenetic manipulation of specific neurons, the bilateral microinjection (300 nL) of the Cre-dependent AAV-expressing channelrhodopsin 2 (ChR2, AAV-DIO-hChR2-mCherry, 2.00 × 10^12^ vg/mL, OBiO Technology Co. Ltd.) in Vglut2-IRES-Cre mice or Cre-dependent AAV-expressing leptosphaeria maculans (MAC, AAV-DIO-MAC-mCherry, 4.00 × 10^12^ vg/mL, OBiO Technology Co. Ltd.), a blue light–sensitive proton pump to enable neural silencing (72) in Vgat-IRES-Cre mice was used to transiently activate glutamatergic neurons or silence GABAergic neurons of the VLO.

To activate the VLO-vlPAG projection pathway, a retrograde virus (AAV2/Retro-EflA-DIO-hChR2-mCherry, 3.8 × 10^12^ vg/mL, 250 nL per side, OBiO Technology Co. Ltd.) was preinjected into the bilateral vlPAG (AP, –4.72 mm; ML, ±0.55 mm; DV, –2.8 mm) of CaMK2A-Cre mice. Also, an anterograde virus (AAV2/9-EflA-DIO-hChR2-mCherry, 2.94 × 10^13^ vg/mL, 300 nL per side, OBiO Technology Co. Ltd.) was injected into the bilateral VLO of CaMK2A-Cre mice.

### Cannula infusion experiment

Mice were anesthetized with sodium pentobarbital (50 mg/kg, i.p.) and placed into a stereotaxic apparatus. A 33-gauge double stainless steel guide cannula (Reward, 62028, RWD Life Science Co.) with double stainless steel stylet plug (Reward, 62128, RWD Life Science Co.) was bilaterally implanted 0.6 mm above the VLO injection site.

The cannula was fixed by tissue glue and dental cement. Then, the animals were returned to their home cage and allowed to recover for 7 days. Microinjection was performed through a 33-gauge double injector cannula (Reward, 62228, RWD Life Science Co.), which protruded 0.6 mm beyond the guide cannula. HCA (DL-Homocysteic acid, Calbiochem), SKF38393, SCH23390, and quinpirole (MilliporeSigma) were dissolved in 0.9% saline. A volume of 0.6 μL per side of either vehicle or drug was injected at the rate of 0.1 μL/min. The injector cannula was left in place for an additional 2 minutes to minimize spread of the drug along the injection track.

### Behavioral experiments

The behavioral experiments described herein were performed by experimenters who were blind to the treatments.

#### von Frey filaments.

Each mouse was handled and habituated in the experimenter’s hand for 30 minutes at least 3 days before testing. Two methods were used to evaluate the mechanical facial response. First, mechanical stimulation was applied by 0.02 and 0.16 g von Frey filaments (Stoelting) within the infraorbital nerve territory. Behavioral response was assessed with the score system described by Vos et al. (73). Second, mechanical sensitivity was determined with a series of von Frey filaments (bending forces: 0.008, 0.02, 0.04, 0.07, 0.16, 0.4, 0.6 g). The filaments was performed in an increasing order from the lowest force, and a touch stimulus force that evoked the positive (fast and obvious) withdrawal response was identified as the mechanical threshold value.

#### OF.

The apparatus consisted of an open box (40 cm × 40 cm × 30 cm, length × width × height). The observation arena was divided into the angle zone (10 cm × 10 cm at the 4 corners), the center of the arena (20 cm × 20 cm), and the periphery zone between the angle and the center (10 cm × 20 cm). The house was maintained in a dim illumination (25 lux) with no noise. Mice were gently placed into the center of the arena and allowed to explore for 5 minutes or 3 minutes (optogenetic experiments). Video tracking software (Ethovision XT v11.5, Noldus BV) was used to record the distance traveled in each zone by the animal.

#### EPM.

The EPM consisted of 4 arms (6 × 35 cm) and a central platform (6 × 6 cm) elevated 50 cm above the floor. Two closed arms were enclosed with 20 cm–high walls crossing with 2 open arms (without walls). The maze was placed in a room with an illumination of 25 lux. Mice were placed in the center of the maze facing an open arm and were allowed to freely explore the maze for 5 minutes or 3 minutes (optogenetic experiments). Both the open/closed arm time and open/closed arm entries were recorded by the video tracking system (Ethovision XT v11.5, Noldus BV).

#### FST.

Mice were placed in a Plexiglas barrel (18 cm diameter, 50 cm height) filled with 10 cm of water at 24°C ± 1°C. Mice were trained for 10 minutes on the first day; their activities a during 5-minute test were recorded by the video in the second test day, and the accumulated immobility time was counted. Immobility was defined as the mouse not making any active movements other than those necessary to keep the head and nose above the water. The animals were dried immediately and returned to their home cages after the test.

#### TST.

The tail suspension box was made of plastic with the dimensions 30 cm × 30 cm × 30 cm (length × width × height). An aluminum suspension pole was positioned in the middle of the top of the box. The mice were suspended in the middle of this apparatus with the tape at 0.1 cm proximal to the tail tip. The distance between the mouse’s nose and the apparatus floor was 2–3 cm. Mice were tested for 6 minutes, and the freezing time in the last 4 minutes was counted. In optogenetic experiments, mice were tested for 5 minutes, and the last 3 minutes was counted. In the sequential 6-minute TST, light was delivered for 3 minutes and was shut off for the next 3 minutes. Then, the animals were taken down from the suspension pole gently and returned to their home cages after the test.

#### SPT.

The SPT was conducted as described previously (74) with slight modifications. The experiment contained 4 stages. The first stage was cage adaptation: The mice were provided with 2 bottles in their home cage, one containing 1% fresh sucrose solution and the other containing regular water. The mice was treated in this way continuously for 48 hours (day 1, 5 p.m., to day 3, 5 p.m.). The second stage was apparatus adaptation: The mice were placed in a homemade black polyethylene apparatus (50 cm × 24 cm × 13 cm, length × width × height) with a transparent polyethylene cover. The apparatus was divided into 5 same-size chambers, and a mouse was placed in each chamber (the black partition prevented the mice from seeing each other, and the holes in the cover allowed them to breathe). Each mouse was provided with 2 tubes (50 mL, Bio-Serv, 9019) in the chamber, one for sucrose solution and the other for regular water. This stage was carried out in an air-conditioned room (23°C ± 1°C) without noise (day 3, 5 p.m., to day 4, 5 p.m.). The third stage was baseline measurement (day 4, 9 p.m., to day 5, 9 a.m., and day 5, 9 p.m., to day 6, 9 a.m.). The test environment was the same as the second stage. The fourth stage was SPT. After the baseline measurement, mice were deprived of both food and water for 12 hours (day 6, 9 a.m., to day 6, 9 p.m.) in their home cages and then moved to the apparatus to test the sucrose preference rate (day 6, 9 p.m., to day 7, 9 a.m.). The bottle or tube position was changed every day, and the placement of the mice in the chamber was random. The preference rate = (sucrose intake/total intake) × 100%.

### Optogenetic tests

One week after injection of optogenetic virus, an LED pin with wireless bipolar optical fiber (Ø200 μm Core, 0.73 NA, 2.4 mm length, 2.0 mm spacing; Hangzhou Newdoon Technology Co. Ltd.) was used, and the optical fiber part was implanted 0.3 mm above the virus injection site. Tissue glue and dental cement were applied to protect the hole of cortical surface and the skull window. Then the animals were allowed to recover for 14 days. Before optogenetic stimulation, the receiver (3.7 g weight, 16.0 mm × 15.6 mm × 10.8 mm [length × width × height]; Hangzhou Newdoon Technology Co. Ltd.), with a Bluetooth-encrypted connection to the controller (Hangzhou Newdoon Technology Co. Ltd.), was positioned through the hole at the tip of the LED pin. Behavioral tests (OF, EPM, TST) were performed with blue light stimulation (473 nm, 5.0 mW, 20 Hz, 25 ms for activation of VLO glutamatergic neurons or a continuous stimulation for inhibition of VLO GABAergic neurons) for 3 minutes.

### Slice preparation and whole-cell patch clamp recordings

Mice were anesthetized with ethyl carbamate (1 g/kg, i.p.) and transcardially perfused with ice-cold cutting solution (20 mL) containing (in mM) 92 NMDG, 2.5 KCl, 1.2 NaH_2_PO_4_, 20 HEPES, 30 NaHCO_3_, 25 glucose, 5 Na-ascorbate, 3 Na-pyruvate, 2 thiourea, 10 MgSO_4_, and 0.5 CaCl_2_ (pH 7.3 with HCl when carbogenated with 95% O_2_ and 5% CO_2_). The brains were quickly removed and cut in coronal brain slices (350 μm) in ice-cold cutting solution using a vibrating microtome (VT1200S, Leica). Slices containing the VLO and rACC were placed in normal artificial cerebrospinal fluid containing (in mM) 119 NaCl, 2.3 KCl, 1 NaH_2_PO_4_, 26.2 NaHCO_3_, 12 glucose, 1.3 MgSO_4_, and 2.5 CaCl_2_ (pH 7.3 when carbogenated with 95% O_2_ and 5% CO_2_, 300–310 mOsm/L) for incubation for 30 minutes. The incubated brain slices were transferred to the recording tank and fixed with a lid net. The CCD imaging system was used to find the VLO and rACC brain areas at low-power lens (×10) and then switched to high-power lens (×60) to find the VLO neurons. Recordings were done using a pipette solution containing (in mM) 125 K-gluconate, 15 KCl, 0.5 EGTA, 10 HEPES, 10 phosphocreatine, 2 Mg-ATP, and 0.5 Na-GTP (pH 7.3, 300–310 mOsm/L).

Whole-cell patch clamp recordings were performed in CaMK2A^+^ pyramidal neurons with a patch clamp amplifier (Axopatch 700B; Axon Instruments), under visual control using differential interference contrast and infrared optics via a water-immersion objective (Olympus BX51WI). We used an Axon 700B amplifier with a Digidata 1550B digitizer (Axon Instruments) to measure APs and resting membrane potential. Patch pipettes (5–10 MΩ) were made of borosilicate glass on a horizontal micropipette puller (P-97, Sutter Instruments). The APs were recorded using the current-clamp mode. Data were collected with pClamp 10.6 software and analyzed.

### Multiple channel electrophysiological recordings in vivo

#### Electrode production.

The electrode consisted of 16 individually insulated nichrome wires (35 μm inner diameter, impedance 300–900 Kohm, Stablohm 675, California Fine Wire), which welded to 18 pin-to-pin connectors (Mil-Max) in a 4,4,4,4 array (wire spacing, 200 μm). The welds were sealed with AB glue, and the exposed nichrome wires were protected with polyethylene glycol 2000.

#### Electrode implantation.

Mice were anesthetized with isoflurane and placed into a stereotaxic apparatus. The 3% isoflurane was used for induction and 1.5% for maintenance during the operation. The skull plane was adjusted to make sure the bregma and lambda were at a horizontal level, then holes were drilled above the VLO on the right (ipsilateral to the CION). Two additional holes were drilled, and screws were embedded to guarantee the implantation of electrode array. A multiwire electrode was implanted unilaterally in VLO (AP, +2.5 mm; ML, +1.0 mm; DV, –1.3 mm), and the implanting site was marked by Neuro-DiI dye stained on the tip of the electrode. The electrode was fixed by tissue glue and dental cement. Then, the mice were carefully transferred to their home cages and allowed to recover for 7 days.

#### The electrophysiological recording.

The multichannel recordings were carried out in a shielding box in a quiet room. In order to reduce the risk of electrode dropping, mice were lightly anesthetized with isoflurane (3%), and the electrode was quickly connected with the recording system. Then, the mice were placed in the shielding box and allowed to move freely for 30 minutes to adapt to the environment. In the TST, the electrical signals of VLO neurons were collected by a multichannel data acquisition system (Zeus, Bio-Signal Technologies). The test lasted 6 minutes in total, and the average firing of VLO neurons during the last 4 minutes was obtained. The sampling frequency was 30 kHz.

Spike duration and firing rate are the most commonly used characteristics to distinguish pyramidal neurons from interneurons (31). In this paper, spike duration was defined as the interval time between peak point and valley point — namely, positive-to-negative-peak duration. The waveform of pyramidal neurons changes slowly on the rise time after the negative wave, so the duration is longer. However, the waveform change of interneurons is rapid and the duration is shorter. For cortical neurons, the firing rate of pyramidal neurons is significantly lower than that of interneurons (31).

Offline Sorter software (Plexon Inc.) was used to filter the Spike Sorting. Data were further analyzed with NeuroExplorer (Nex Technologies) and MATLAB to obtain the changes of firing frequency of VLO neurons.

### Statistics

The data are presented as the mean ± 95% CI. No statistical power calculation was conducted before the study. The sample sizes were based on our previous knowledge and experience with this design. Mice that showed hyperactivity and lethargy in behavioral tests were excluded from the experiments. Comparisons between 2 groups were performed using a 2-sided Student’s *t* test or paired *t* test. Comparisons between 3 or more groups were performed using 1-way, 2-way, or 2-way RM ANOVA followed by post hoc Student-Newman-Keuls test. *P* < 0.05 was considered statistically significant. The statistical analyses were performed using GraphPad Prism 7.0 software.

### Study approval

All experimental procedures were approved by the Experimental Animal Ethics Committee of Shanghai Medical College and the Committee on the Use of Animal Experiments of Fudan University (permit SYXK2009-0082).

## Author contributions

HYS and YQZ designed the project. HYS, YQC, and TTL performed the behavioral experiments. SSL and WL conducted the in vitro whole-cell patch clamp recordings. WS and LZ conducted the multiple-channel electrophysiological recordings in vivo and data analysis. NL and HC contributed to the preparation of experimental apparatus and medicines. YQZ supervised the project. HYS and YQZ analyzed data and wrote the paper.

## Supplementary Material

Supplemental data

Supplemental Video 1

Supplemental Video 2

Supplemental Video 3

## Version 1 10/02/2020

Electronic publication
