# Supplementary material for: Activation of ventrolateral orbital cortex improves mouse neuropathic pain–induced anxiodepression
Source: JCI Insight. 2020 Oct 2;5(19):e133625. doi: 10.1172/jci.insight.133625 (PMC7566721; doi:10.1172/jci.insight.133625)
Supplement: Supplemental data [file jciinsight-5-133625-s063.pdf]

Supplemental Information

Activation of ventrolateral orbital cortex improves mouse neuropathic pain-induced anxiodepression

Hai-Yan Sheng[1,2], Su-Su Lv[1], Ya-Qi Cai[1], Wu Shi[3], Wei Lin[1], Ting-Ting Liu[1], Ning Lv[1], Hong Cao[1], Ling Zhang[3], Yu-Qiu Zhang[1*]

[1]State Key Laboratory of Medical Neurobiology and MOE Frontiers Center for Brain Science, Department of Translational Neuroscience, Jing'an District Centre Hospital of Shanghai, Institutes of Brain Science, Fudan University, Shanghai 200032, China.

[2]Department of Pathophysiology, School of Basic Medical Sciences, Xinxiang Medical University, Henan 453003, China.

[3]The First Rehabilitation Hospital of Shanghai, Tongji University School of Medicine, Shanghai 200090, China.

Inventory of Supplemental Information

Figure S1. related to Figure 2, Excitation of the bilateral VLO induced anti-anxiodepressive effect in TN mice.

Figure S2. related to Figure 2, Chemogenetical activation of bilateral VLO neurons did not change the anxiety/depression levels in naive mice.

Figure S3. related to Figure 3, Chemogenetical inhibition of bilateral VLO neurons did not change the anxiety/depression levels in naive mice.

Figure S4. related to Figure 3, Chemogenetical inhibition of bilateral VLO CaMK2A+ neurons did not change the anxiety/depression level in naive mice.

Figure S5. related to Figure 4, Membrane properties of VLO and rACC CaMK2A+ neurons and spontaneous firings of VLO pyramidal neurons.

Figure S6. related to Figure 9 and Figure 10, Activation of Sm-VLO and VLO-vlPAG projections inhibited TN-induced mechanical allodynia.

Movi S1. related to Figure 1, TN mice exhibits longer freezing time in TST than sham one.

Movi S2. related to Figure 6, Optogenetic activation of VLO glutamatergic neurons shortens the freezing time in TST .

Movi S3. related to Figure 7, Optogenetic inhibition of VLO GABAergic neurons shortens the freezing time in TST.

Supplemental Figures

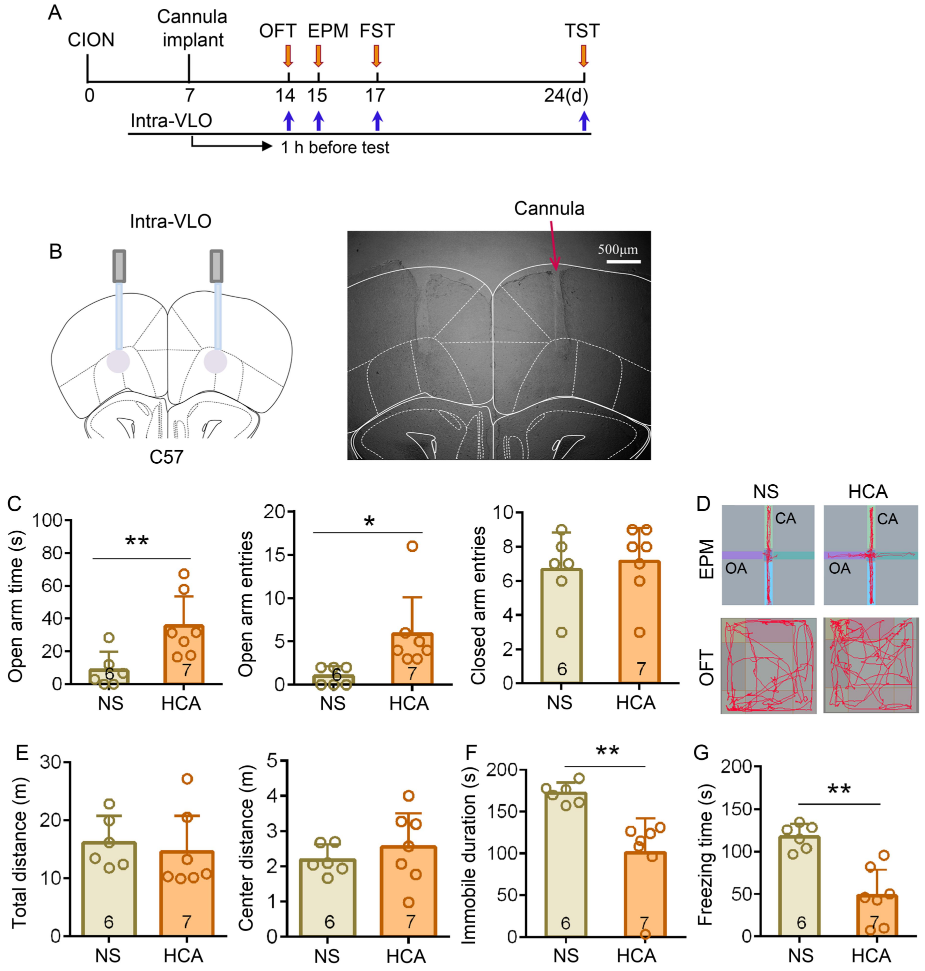

Figure S1. related to Figure 2, Excitation of the bilateral VLO induced anti-anxiodepressive effect in TN mice. (A) Schematic of the protocol for experiments C-G. (B) Schematic and photomicrograph of coronal section showing cannula placement in the bilateral VLO in mice. Scale bar: 500 µm. (C-G) Microinjection of HCA (DL-Homocysteic acid, 30 nmol per side), a glutamate replacement, into the bilateral VLO led to a significant anti-anxiodepressive effect in EPM and OFT (C-E), FST (F) and TST (G) in TN mice, *p<0.05, **p<0.01, two-sided Student's t-test, n=6 and 7.

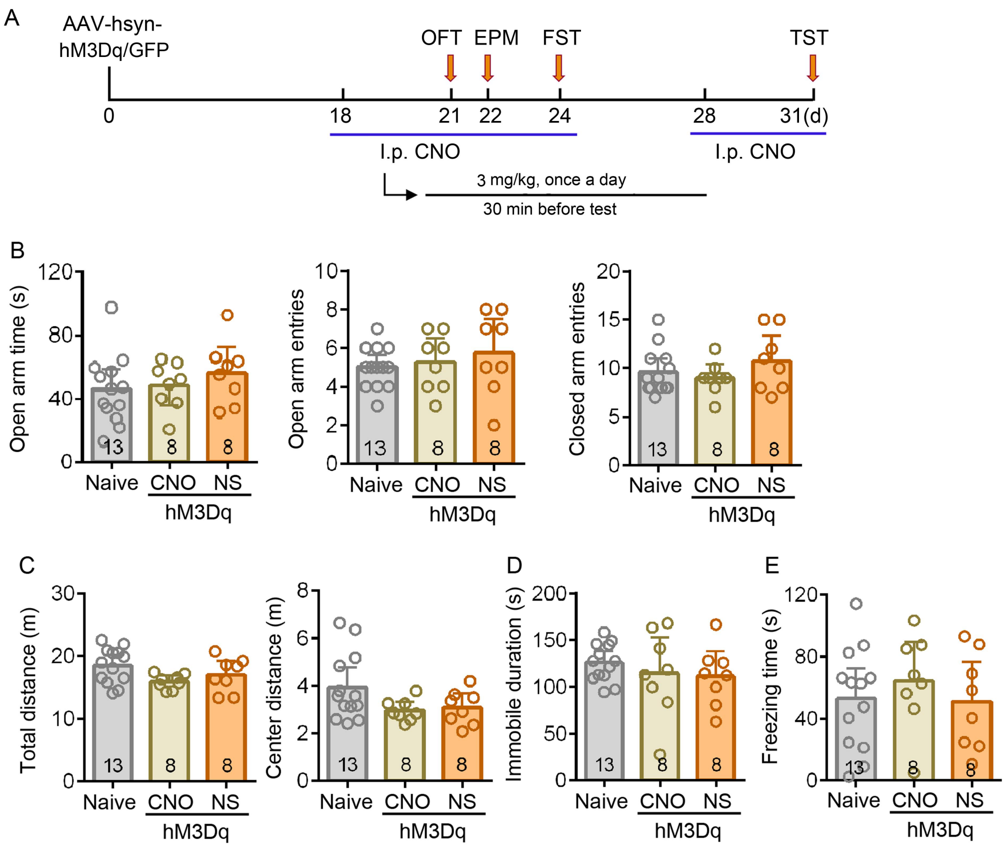

Figure S2. related to Figure 2, Chemogenetical activation of bilateral VLO neurons did not change the anxiety/depression levels in naive mice. (A) Schematic of the protocol for experiments B-E. (B-E) There were no differences in behaviors of EPM (B), OFT (C), FST (D), TST (E) among the naive, intra-VLO of AAV-hsyn-hM3Dq-GFP with i.p. CNO (500 nM) and intra-VLO of AAV-hsyn-hM3Dq-GFP with i.p. normal saline (NS) groups, one-way ANOVA, n=13, 8, 8.

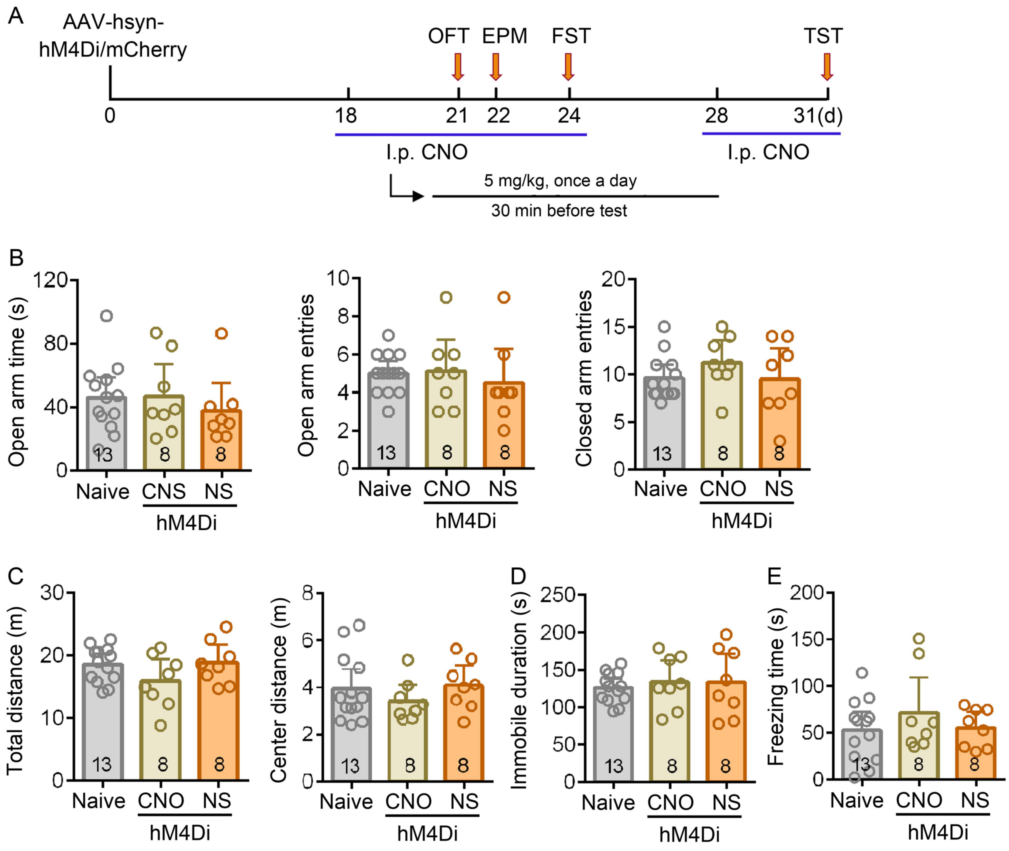

Figure S3. related to Figure 3, Chemogenetical inhibition of bilateral VLO neurons did not change the anxiety/depression levels in naive mice. (A) Schematic of the protocol for experiments B-E. (B-E) There were no differences in behaviors of EPM (B), OFT (C), FST (D), TST (E) among the naive, intra-VLO of AAV-hsyn-hM4Di-mCherry with i.p. CNO (500 nM) and intra-VLO of AAV-hsyn-hM4Di-mCherry with i.p. normal saline (NS) groups, one-way ANOVA, n=13, 8, 8.

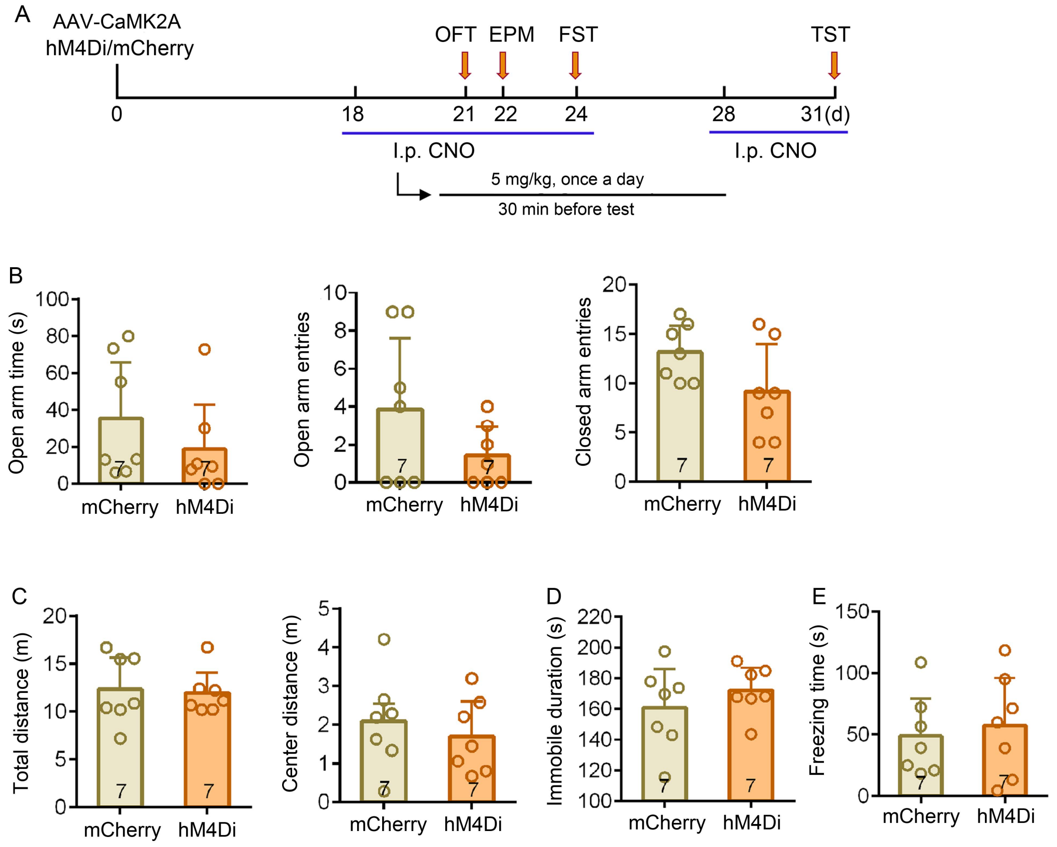

Figure S4. related to Figure 3, Chemogenetical inhibition of bilateral VLO CaMK2A+ neurons did not change the anxiety/depression level in naive mice. (A) Schematic of the protocol for experiments B-E. (B-E) There were no differences in behaviors of EPM (B), OFT (C), FST (D), TST (E) between the intra-VLO of AAV-CaMK2A-hM4Di-mCherry and AAV-CaMKIIα-mCherry groups, two-sided Student's t-test, n=7.

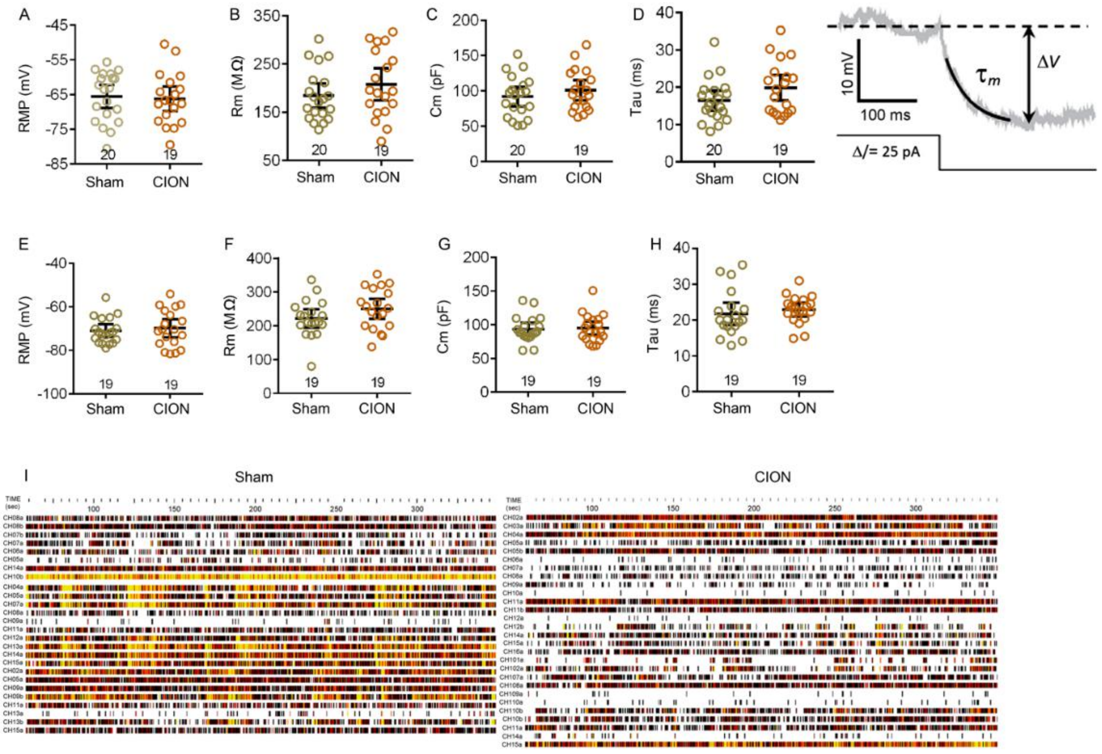

Figure S5. related to Figure 4, Membrane properties of VLO and rACC CaMK2A+ neurons and spontaneous firings of VLO pyramidal neurons. (A-D) Resting membrane potential (RMP, A), input resistance (B), membrane capacitance (C) and time constant (Tau) (D) of CaMK2A+ pyramidal neurons in the VLO from the mice 14 days after sham and CION. Two-sided Student's t-test, n=20 and 19. (E-H) Resting membrane potential (RMP, E), input resistance (F), membrane capacitance (G) and time constant (Tau) (H) of CaMK2A+ pyramidal neurons in the rACC from the mice 14 days after sham and CION. Two-sided Student's t-test, n=19. (I) The spontaneous firing rate of 28 VLO pyramidal neurons from 3 CION mice and 26 pyramidal neurons from 4 sham mice.

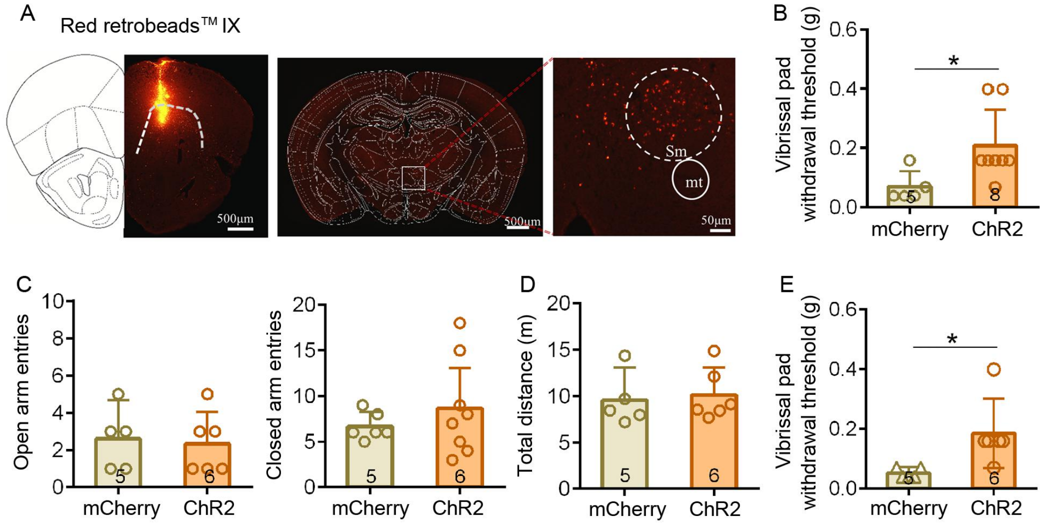

Figure S6. related to Figure 9 and Figure 10, Activation of Sm-VLO and VLO-vlPAG projections inhibited TN-induced mechanical allodynia. (A) Fluorescent RetroBeads IX was injected into unilateral VLO, and the fluorescent signaling was detected in the ipsilateral submedius thalamic nucleus (Sm). Scale bar: 500 μm for the left and middle, 50 μm for the right. (B) Chemogenetic activation of Sm-VLO projection pathway alleviated TN-induced mechanical allodynia. *$p<0.05$, two-sided Student's t-test, n=5 and 8. (C and D) Optogenetic activation of the VLO-vlPAG projection had no effect on anxiety-like behaviors and locomotor acivity in EPM (C) and OFT (D). (E) Optogenetic activation of the VLO-vlPAG excitatory projection attenuated TN-induced allodynia. *$p<0.05$, two-sided Student's t-test, n=5 and 6.
